# Ethnic differences in the severity and clinical management of type 2 diabetes at time of diagnosis: A cohort study in the UK Clinical Practice Research Datalink

**DOI:** 10.1016/j.diabres.2020.108006

**Published:** 2020-02

**Authors:** R. Mathur, L. Palla, R.E. Farmer, N. Chaturvedi, L. Smeeth

**Affiliations:** a London School of Hygiene & Tropical Medicine, Department of Non-Communicable Disease Epidemiology, Keppel Street, London WC1E 7HT, UK; b University College London, Institute of Cardiovascular Sciences, Gower Street, London WC1E 6BT, UK

**Keywords:** Type 2 diabetes, Ethnicity, Epidemiology, Inequalities, Primary care, Treatment

## Abstract

**Aims:**

To characterize ethnic differences in the severity and clinical management of type 2 diabetes at initial diagnosis.

**Methods:**

An observational cohort study of 179,886 people with incident type 2 diabetes between 2004 and 2017 in the Clinical Practice Research Datalink was undertaken; 63.4% of the cohort were of white ethnicity, 3.9% south Asian, and 1.6% black. Ethnic differences in clinical profile at diagnosis, consultation rates, and risk factor recording were derived from linear and logistic regression. Cox-proportional hazards regression was used to determine ethnic differences in time to initiation of therapeutic and non-therapeutic management following diagnosis. All analyses adjusted for age, sex, deprivation, and clustering by practice.

**Results:**

In the 12 months prior to diagnosis, non-white groups had fewer consultations compared to white groups, but risk factor recording was better than or equivalent to white groups for 9/10 risk factors for south Asian groups and 8/10 risk factors for black groups (p < 0.002). Blood pressure, BMI, cholesterol, eGFR, and CVD risk levels were more favourable in non-white groups, and prevalence of macrovascular disease was significantly lower (p < 0.003). Time to initiation of antidiabetic treatment and first risk assessment was faster in non-white groups relative to white groups, while time to risk factor measurement and diabetes review was slower.

**Conclusions:**

We find limited evidence of systematic ethnic inequalities around the time of type 2 diabetes diagnosis. Ethnic disparities in downstream consequences may relate to genetic risk factors, or manifest later in the care pathway, potentially in relation to long-term risk factor control.

## Highlights

• Non-white groups had better or equivalent capture of risk factors prior to diagnosis compared to white groups.
• Risk factor levels at diagnosis were more favourable for south Asian and Black groups.
• Initiation of diabetes therapy was faster for non-white groups relative to white groups.
• Downstream inequalities in type 2 diabetes do not appear to stem from inequalities in initial diagnosis.

## 1 Introduction

Marked ethnic differences in the risk of long-term vascular outcomes among people with type 2 diabetes have been established in UK populations [1], [2]. The extent to which these inequalities stem from modifiable factors such as healthcare usage or quality of diabetes management remains unclear. Given that inequalities can accumulate over time, it is vital to identify where along the care pathway differences by ethnicity may arise. Though equity of service provision is a central tenet of the National Health Service (NHS) [3], recent studies have highlighted ethnic differences in access to healthcare, treatment provision and risk factor control [4], [5], [6], [7], [8].

In the UK, the collection of ethnicity data via official statistics has been mandated since the Race Relations Act of 1968 as a vital first step towards identifying and actively tackling ethnic inequalities. The 16 ethnic group categories defined by the 2001 Census for England and Wales currently form the national standard for mandatory ethnicity data collection across the National Health Service. Ethnicity is self-reported by individuals at either initial registration with their general practitioner or during consultation, and intended to reflect the individuals own self-perception of belonging to, or identifying with a certain social group [9], [10].

Prior to initial diagnosis, there may be differences by ethnicity in consultation rates and measurement of risk factors, which may impact upon the timeliness of diagnosis and severity of disease at initial presentation. Delays in diagnosis may result in delays in initiation of therapeutic and non-therapeutic management, which may further compound existing inequalities. Though guidelines exist for managing type 2 diabetes in the UK, the extent to which these are followed may differ by ethnic group, leading to inequalities in the downstream consequences of type 2 diabetes [8], [11]. The 2018 UK national diabetes audit identified inequalities by age, region, diagnosed serious mental illness and learning disabilities, but did not explore differences by ethnicity, leaving a critical gap in the evidence base [12].

The aims of this study were to (1) Quantify ethnic differences in risk factor levels and co-morbidities at the time of initial diagnosis, (2) Compare consultation rates and completeness of process of care measures between ethnic groups in the 12 months preceding type 2 diabetes diagnosis, (3) Determine whether the time to initiation of therapeutic and non-therapeutic management following initial diagnosis differed by ethnic group.

## 2 Methods

### 2.1 Study design and population

An observational cohort study utilizing the Clinical Practice Research Datalink (CPRD) was undertaken. The CPRD is a clinical research database containing anonymised longitudinal primary care records for approximately 15 million people from 714 general practices. The CPRD population has been shown to be representative of the UK population with respect to age, gender, and ethnicity [13].

Type 2 diabetes was identified using an adjudication algorithm developed to minimize misclassification of diabetes status and type in electronic health records [14]. Briefly, the algorithm assigns an initial diabetes type based on clinical Read codes [15] – C10E for type 1 diabetes and C10F for type 2 diabetes- and then applies a series of logic rules to assign a final diabetes status by identifying congruent or contradictory evidence on age at diagnosis, diabetes medications (excluding individuals on metformin only as this may be indicated for conditions other than type 2 diabetes such as polycystic ovarian syndrome or pre-diabetes), hyperglycaemia (HbA1c ≥ 6.5% or 48 mmol/mol, or fasting/ random/ unspecified glucose ≥ 11.1 mmol/l) and presence of diabetes process of care codes. For individuals with a prescription for antidiabetic medication in the 12 months preceding the first ever type 2 diabetes diagnosis, the diagnosis date was moved forward to the date of prescription as it was deemed plausible that the prescription was related to the initial diagnosis. As misclassification of prevalent diagnoses as incident diagnoses is more likely around the time of initial registration with the general practitioner, a minimum registration period of six months prior to initial diagnosis of type 2 diabetes was required [16]. Adults aged 18 and over registered between 2004 and 2017, with at least six months of continuous registration prior to diagnosis of type 2 diabetes (the earliest of diagnosis date or medication date where applicable) were included in the study.

### 2.2 Covariates

Self-reported ethnicity, identified using Read codes, was collapsed into the five categories of the 2001 UK census (white, south Asian, black African/Caribbean, mixed, and other). For individuals with more than one ethnicity code on their primary care record, a previously developed algorithm was used to assign a best ‘single’ ethnicity – based on the most commonly, and most recently recorded codes (Supplementary material, Figure S1) [17]. Age at diagnosis was calculated by subtracting year of birth from year of diagnosis. Deprivation was measured using quintiles of the 2015 Index of Multiple Deprivation (IMD) – a measure of small area deprivation based on an individual’s home postcode [18]. For people with linkage to Office for National Statistics data, quintiles of IMD were derived from the individual’s home postcode. For the 40% of people without linkage, quintiles were derived from the postcode of the individual’s general practice.

Baseline risk factors were identified from the most recently recorded value in the 12 months preceding type 2 diabetes diagnosis (see supplementary table S3 for all code lists). These included glycated haemoglobin (HbA1c), fasting blood glucose (FBG), systolic and diastolic blood pressure (SBP, DBP), body mass index (BMI), total cholesterol, serum creatinine, consultations (face-to-face or telephone), smoking status (‘Ever smoker’, and ‘Never smoker’), alcohol consumption (‘Non-drinker’, ‘Moderate drinker’, and ‘Heavy drinker’), and family history of cardiovascular disease (CVD). Risk assessments included ten-year CVD risk and the NHS health check. The CVD risk score, was categorized into “≤ 10% risk of developing CVD in the next ten years” and “>10% risk of developing CVD in the next ten years”.

Pre-diabetic states included coded pre-diabetes, family history of diabetes, and gestational diabetes (for women only). Co-morbidities were considered present at baseline if recorded at any time prior to diagnosis. Macrovascular co-morbidities included hypertension, coronary heart disease (CHD, including myocardial infarction and angina), stroke, and heart failure. Microvascular co-morbidities included chronic kidney disease (CKD), retinopathy, and neuropathy.

To examine diabetes management following initial diagnosis, the date of the first antidiabetic medication prescription (including oral antidiabetic agents and insulin), consultation, risk factor measurement, diabetes review (including retinopathy screening, foot examination, and offer of dietary advice), offer of structured diabetes education, and risk assessment following diagnosis was extracted.

### 2.3 Statistical analysis

As individuals attending the same general practice may have similar levels of care provision and clinical coding, multilevel modelling was used to account for the clustering of people within practices. Ethnic differences in clinical characteristics at diagnosis were derived from multilevel multivariable linear regression, (for age at diagnosis, HbA1c, FBG, SBP, DBP, BMI, total cholesterol, serum creatinine, and eGFR) and multilevel multivariable logistic regression (for deprivation quintile, presence of pre-diabetes, family history of diabetes, gestational diabetes, family history of cardiovascular disease, any macrovascular disease, any microvascular disease, smoking status, alcohol consumption, CVD risk, and use of antihypertensive or lipid lowering drugs) and adjusted for age at diagnosis, sex, and deprivation. Multilevel multivariable logistic regression adjusted for age at diagnosis, sex, and deprivation was used to determine ethnic differences in the odds of having each risk factor recorded in the 12 months prior to diagnosis. Multivariable Cox-proportional hazards regression adjusting for age at diagnosis, sex, deprivation, raised HbA1c at baseline (>7.5%/53 mmol/L), and clustering by practice was used to compare time to initiation of therapeutic and non-therapeutic diabetes management between ethnic groups. Follow-up time began at the date of type 2 diabetes onset and ended at the earliest of: first antidiabetic prescription or care process, leaving the CPRD, last data collection, or death.

### 2.4 Sensitivity analysis

We conducted a sensitivity analysis comparing outcomes for those of unknown ethnicity to those of any known ethnicity. As the recording of ethnicity was financially incentivised under the Quality and Outcomes Framework from 2006 to 2011, and forms a core component of the annual NHS Health Check [19] and the NHS diabetes management guidelines [20], the recording of ethnicity can be considered a marker of engagement with primary care. We hypothesized that individuals with missing ethnicity would have poorer risk factor control at diagnosis, lower consultation rates, worse capture of risk factors prior to diagnosis and slower initiation of therapeutic and non-therapeutic management relative to those with ethnicity recorded.

## 3 Results

**Fig. 1:**
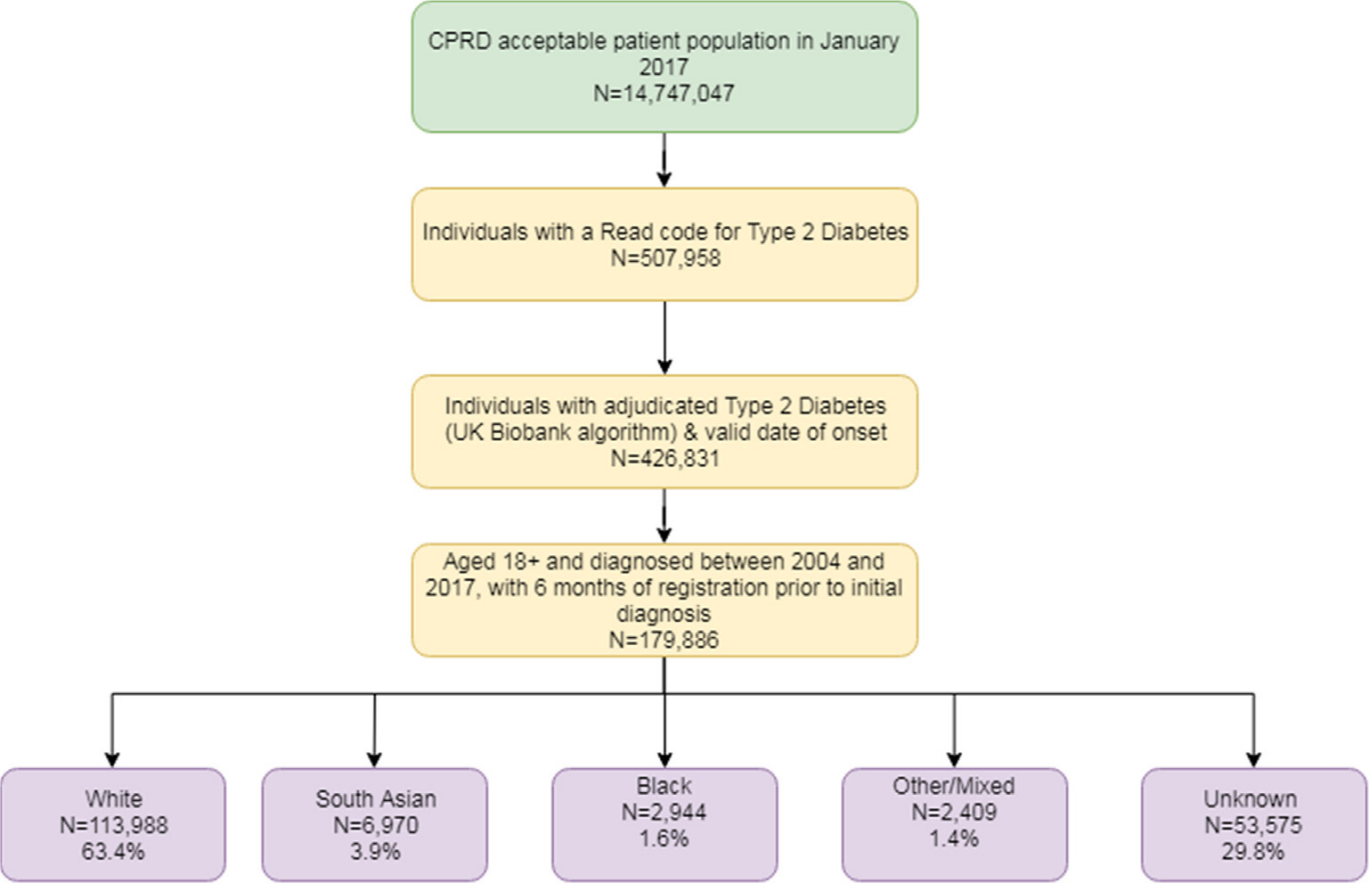
Study population flowchart.

From 241,891 individuals diagnosed with type 2 diabetes between April 1st, 2004 and December 31st, 2016 in the CPRD, 179,886 adults aged 18 or over, with at least 6 months registration prior to initial diagnosis, were included in the study (Fig. 1). Within this population, 5% (n = 8871) had been prescribed an antidiabetic medication in the year prior to diagnosis and had their diagnosis date moved backwards. Ethnicity was recorded for 70% of the cohort (n = 126,331), of whom 90.2% were white (n = 113,988), 5.5% were south Asian (n = 6970), 2.3% were black African/Caribbean (n = 2944), and 1.9% were of other ethnicities, including mixed (n = 2409). Comparisons between the white, south Asian and black ethnic groups are reported below.

### 3.1 Clinical characteristics at initial diagnosis

**Fig. 2:**
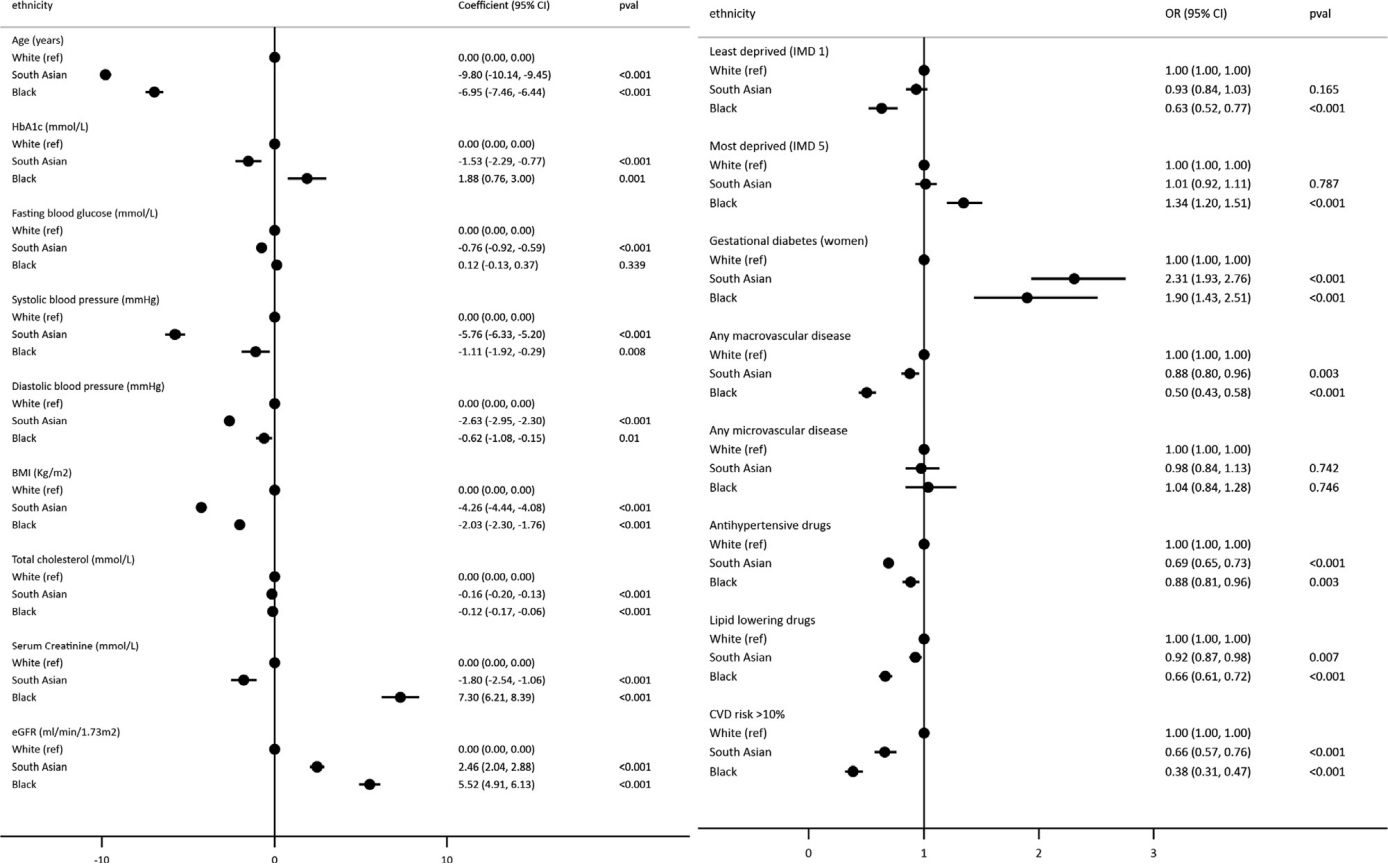
Ethnic differences in clinical severity at type 2 diabetes diagnosis. *All models adjust for age at diagnosis, sex, deprivation, and clustering by practice.

Crude ethnic differences in clinical characteristics at initial diagnosis are described in Table 1 and adjusted differences are illustrated in Fig. 2. After adjustment for sex, deprivation, calendar year and clustering by practice, age at diagnosis was estimated to be 9.8 years earlier in south Asian groups than white groups (95%CI −10.14, −9.45) and 7 years earlier in black groups (95%CI −7.46, −6.44) relative to white and 7 years earlier in black groups (data) relative to white groups. Black groups were overrepresented in the least affluent deprivation quintile compared to white groups (OR 1.34, 95%CI 1.20–1.51), while no differences in deprivation between white and south Asian groups were evident.

**Table 1:** Ethnic differences in clinical characteristics at time of initial Type 2 diabetes diagnosis.

		% complete	White	South Asian	Black	Other	Mixed	Unknown
*N*			113,988	6970	2944	1854	555	53,575
**Demographic characteristics**	Age at diagnosis (mean, SD)	100.0	63.2 (13.4)	52.6 (13.1)	55.1 (13.1)	56.3 (13.2)	54.6 (13.6)	62 (13.4)
	Male, %	100.0	62,810 (55.1)	3767 (54)	1498 (50.9)	1022 (55.1)	304 (54.8)	30,643 (57.2)
	Social Deprivation, %	100.0						
	Quintile 1 (Least deprived)		20,889 (18.3)	880 (12.6)	169 (5.7)	302 (16.3)	86 (15.5)	11,443 (21.4)
	Quintile 2		22,486 (19.7)	1081 (15.5)	264 (9)	262 (14.1)	87 (15.7)	8335 (15.6)
	Quintile 3		25,343 (22.2)	1456 (20.9)	611 (20.8)	368 (19.8)	120 (21.6)	9973 (18.6)
	Quintile 4		22,278 (19.5)	1605 (23)	820 (27.9)	477 (25.7)	117 (21.1)	13,255 (24.7)
	Quintile 5 (Most deprived)		22,992 (20.2)	1948 (27.9)	1080 (36.7)	445 (24)	145 (26.1)	10,569 (19.7)

**Health behaviours**	Smoking status, %	72.1						
	Non-Smokers		32,098 (38.3)	3106 (70.6)	1227 (65.2)	683 (54.6)	189 (49.6)	16,406 (43.2)
	Current Smokers		18,479 (22)	701 (15.9)	288 (15.3)	258 (20.6)	100 (26.2)	8037 (21.2)
	Ex-Smokers		33,282 (39.7)	593 (13.5)	367 (19.5)	311 (24.8)	92 (24.1)	13,536 (35.6)

**Risk factor level (mean, SD)**	HbA1c, mmol/L	52.9	63.2 (22.9)	63.9 (22.7)	66.3 (25)	65 (23.5)	66.6 (24.3)	65.2 (23)
	HbA1c, %	52.9	7.9 (2.1)	8 (2.1)	8.2 (2.3)	8.1 (2.2)	8.2 (2.2)	8.1 (2.1)
	Fasting blood glucose	66.4	10.5 (5)	10.1 (4.7)	10.7 (5.6)	9.9 (4.5)	10.6 (5.3)	10.7 (5.1)
	SBP, mmHg	88.1	142.9 (19.7)	135.5 (18.7)	140.3 (19.5)	138.2 (19.6)	139.2 (20.5)	143.4 (20.2)
	DBP, mmHg	88.1	82.3 (11.6)	82.3 (11.3)	83.8 (11.4)	82.5 (11)	84 (11.7)	82.9 (11.8)
	BMI, Kg/m2	63.6	32.3 (6.1)	29.7 (5.3)	31.8 (5.9)	30.1 (5.9)	31.3 (6.4)	32.3 (6.1)
	Total cholesterol, mmol/L	78.2	5.2 (1.2)	5.3 (1.2)	5.3 (1.1)	5.3 (1.2)	5.4 (1.2)	5.3 (1.2)
	Serum creatinine, mmol/L	87.0	86.7 (28.6)	78.6 (27.7)	88.5 (42.3)	79.2 (27)	82.4 (25.4)	85.1 (25.9)
	ACR, mg/mmol	2.5	19.6 (46.7)	20.5 (44.3)	15.7 (25.3)	38.1 (70.7)	12.9 (10.8)	20.2 (51.7)

**CVD risk score (%)**	>10% risk in 10 years	17.6	79.1	58.4	50.8	64	53.6	78.3
**Pre-diabetic indicators**+	Pre-diabetes		19,007 (16.7)	1310 (18.8)	496 (16.8)	319 (17.2)	96 (17.3)	8693 (16.2)
	Family history of diabetes		14,471 (12.7)	1916 (27.5)	659 (22.4)	350 (18.9)	127 (22.9)	5941 (11.1)
	Gestational diabetes*		784 (1.5)	237 (7.4)	72 (5)	41 (4.9)	15 (6)	293 (1.3)
	Family history of CVD		50,813 (44.6)	2874 (41.2)	858 (29.1)	660 (35.6)	212 (38.2)	23,305 (43.5)

**Diagnosed co-morbidities (%)**+	Any macrovascular disease		22,122 (19.4)	720 (10.3)	211 (7.2)	184 (9.9)	49 (8.8)	8975 (16.8)
	Any microvascular disease		4781 (4.2)	217 (3.1)	101 (3.4)	49 (2.6)	14 (2.5)	1994 (3.7)
	Hypertension		57,625 (50.6)	2320 (33.3)	1375 (46.7)	748 (40.3)	222 (40)	26,000 (48.5)
	CHD		19,260 (16.9)	649 (9.3)	138 (4.7)	162 (8.7)	44 (7.9)	7746 (14.5)
	Stroke		507 (0.4)	12 (0.2)	8 (0.3)	9 (0.5)	0.0	204 (0.4)
	Heart failure		5117 (4.5)	133 (1.9)	90 (3.1)	33 (1.8)	8 (1.4)	2253 (4.2)
	CKD		708 (0.6)	24 (0.3)	16 (0.5)	6 (0.3)	3 (0.5)	267 (0.5)
	Retinopathy		2111 (1.9)	106 (1.5)	56 (1.9)	26 (1.4)	9 (1.6)	979 (1.8)
	Neuropathy		2215 (1.9)	101 (1.4)	38 (1.3)	20 (1.1)	2 (0.4)	845 (1.6)

**Medications prescribed (%)**+	Antihypertensives		69,988 (61.4)	2796 (40.1)	1441 (48.9)	838 (45.2)	258 (46.5)	31,095 (58)
	Lipid lowering		52,721 (46.3)	2566 (36.8)	926 (31.5)	729 (39.3)	190 (34.2)	22,834 (42.6)

* Baseline covariate data taken at the date closest to Type 2 diabetes diagnosis in the 12 months preceding diagnosis, (gestational diabetes among women only).

+ Pre-diabetic indicators, Diagnosed co-morbidities and medications assumed to be present if recorded and absent if not recorded.

After additionally accounting for age at diagnosis, mean HbA1c was lower in south Asian groups

(β = −1.53, 95%CI −2.29, −0.77) and higher in black groups relative to white groups, (β = 1.88 95%CI 0.76–3.00). BMI, total cholesterol, and eGFR were more favourable in non-white groups compared to white groups at diagnosis (p < 0.001), while fasting blood glucose, blood pressure and creatinine levels were better for south Asian groups only (p < 0.034). The odds of having co-morbid macrovascular disease at diagnosis were reduced in south Asian groups and halved in black groups relative to white (South Asian OR 0.88, 95%CI 0.80–0.96, Black OR 0.50, 9%CI 0.43–0.58); no ethnic differences in the odds of having diagnosed microvascular disease were evident. Furthermore, non-white groups had markedly fewer prescriptions of antihypertensive and lipid lowering drugs in the 12 months preceding diagnosis, and reduced odds of having a CVD risk score over 10% relative to white groups (p < 0.007) (Fig. 2).

### 3.2 Clinical management prior to diagnosis

In the 12 months prior to diagnosis, consultation frequency was higher for white groups (median 10, IQR 6–17) than for south Asian (median 9, IQR 5–15) and black groups (median 8, IQR 5–14). After adjustment for age at diagnosis, sex, deprivation, and clustering by practice, the consultation rate was significantly lower for black groups relative to white (β = −0.60, 95%CI −1.05, −0.21). Risk factor recording for south Asian groups was better than or equivalent to non-white groups for 9/10 risk factors of interest, and for black groups, risk factor recording was better or equivalent for 8/10 risk factors (Table 2).

**Table 2:** Risk factor recording and consultations in the 12 months prior to type 2 diabetes diagnosis.

	% with risk factor recorded			Adjusted difference					
	White	South Asian	Black	South Asian vs. White			Black vs. White		
N	113,988	6970	2944						
Risk Factors	%	%	%	OR	95%CI	p.val	OR	95%CI	p.val
HbA1c	51.8	62.3	60.9	1.37	(1.30, 1.45)	<0.001	1.17	(1.08, 1.27)	<0.001
Glucose	66.2	60.8	59.4	1.02	(0.97, 1.08)	0.455	0.88	(0.81, 0.95)	0.002
Blood Pressure	88.9	85.0	87.0	0.99	(0.92, 1.06)	0.700	1.08	(0.97, 1.19)	0.162
BMI	64.1	64.6	64.6	0.98	(0.93, 1.03)	0.345	1.00	(0.92, 1.08)	0.950
Total Cholesterol	78.9	80.8	78.6	1.12	(1.06, 1.19)	<0.001	1.01	(0.92, 1.09)	0.892
Creatinine	87.7	85.3	84.9	1.14	(1.07, 1.21)	<0.001	1.07	(0.97, 1.17)	0.163
Urine ACR	2.4	1.9	2.1	1.18	(0.96, 1.44)	0.117	1.18	(0.89, 1.57)	0.242
Smoking Status	73.6	63.1	63.9	0.72	(0.69, 0.76)	<0.001	0.75	(0.70, 0.81)	<0.001

Risk assessments									
NHS Health Check	4.1	8.4	10.2	1.55	(1.40, 1.73)	<0.001	1.49	(1.29, 1.72)	<0.001
CVD risk score	18.7	22.3	24.9	1.05	(0.98, 1.12)	0.159	1.22	(1.11, 1.35)	<0.001

Consultations	Median (IQR)			β	CI95%	p. val	β	CI95%	p. val
Number of consultations	10 (6–17)	9 (5–15)	8 (5–14)	−0.10	(−0.40, 0.18)	0.452	−0.60	(−1.05, −0.21)	0.003

*Logistic and linear regression models adjusted for age at baseline, sex, deprivation, and clustering by practice.

### 3.3 Initiation of therapeutic and non-therapeutic management following diagnosis

After adjustment for age, sex, deprivation, baseline HbA1c, and clustering by practice, time to initiation of antidiabetic therapy was faster south Asian groups (HR 1.10, 95%CI 1.07–1.14) and black groups relative to white (HR 1.18, 95%CI 1.12–1.23). Time to first NHS health check (South Asian HR 1.30, 95%CI 1.10–1.54, Black HR 1.33, 95%CI 1.6–1.68) and offer of structured diabetes education (South Asian HR 1.17, 95%CI 1.10–1.24, Black HR 1.44, 95%CI 1.31–1.56) was also faster in south Asian and black groups relative to white. In contrast, time to first consultation, risk factor measurement and diabetes review was longer or equivalent for both non-white groups relative to white (Table 3).

**Table 3:** Time to therapeutic and non-therapeutic clinical management following type 2 diabetes diagnosis.

	% receiving clinical management			Median time to first clinical event (months)			Adjusted HR					
	White	South Asian	Black	White	South Asian	Black	SA vs. White			Black vs. White		
N	113,988	6970	2944				HR	CI95%	p.val	HR	CI95%	p.val
First post-diagnosis consultation	99.9	99.7	99.8	0.1	0.1	0.1	0.84	(0.81,0.86)	<0.001	0.92	(0.89,0.96)	<0.001

Initiation of antidiabetic therapy	73.2	80.9	78	3.6	2.2	1.8	1.10	(1.07,1.14)	<0.001	1.18	(1.12,1.23)	<0.001

Risk factor measurement												
HbA1c	94.8	92.4	89.7	2.7	3.2	3.2	0.93	(0.91,0.96)	<0.001	0.88	(0.84,0.91)	<0.001
Blood Glucose	61.7	58.1	56.2	10.5	10.3	9.2	0.94	(0.91,0.98)	0.001	0.96	(0.91,1.01)	0.125
Urine ACR	59	53.4	56.4	11.4	12.2	10.6	1.01	(0.98,1.05)	0.504	1.05	(1.00,1.11)	0.055
BMI	92.7	91.2	88.6	1.7	2.1	2.4	0.96	(0.94,0.99)	0.010	0.97	(0.93,1.01)	0.115
Blood Pressure	96.3	94.2	93.5	1.4	1.6	1.4	0.91	(0.88,0.93)	<0.001	1.00	(0.96,1.04)	0.872
Total Cholesterol	92.5	90.2	87.6	3.7	4.3	4.1	0.99	(0.97,1.02)	0.667	0.97	(0.93,1.02)	0.220
Smoking Status	93.2	90.4	86.6	3.0	3.4	3.6	0.90	(0.87,0.92)	<0.001	0.87	(0.83,0.91)	<0.001
Serum Creatinine	94.3	90.7	88.3	3.2	4.0	3.8	0.94	(0.92,0.97)	<0.001	0.93	(0.89,0.97)	<0.001

Diabetes review												
Diabetes Review	82.4	81.1	75.9	6.0	6.7	6.8	1.03	(1.00,1.06)	0.076	1.03	(0.98,1.08)	0.194
Retinopathy Screen	41.1	37.9	41.8	22.8	22.1	19.2	0.98	(0.94,1.02)	0.305	0.93	(0.87,0.98)	0.014
Foot Examination	40	26.9	25.7	26.5	29.8	25.8	0.86	(0.81,0.90)	<0.001	0.92	(0.85,0.99)	0.027
Offer of dietary advice	6.3	3.3	1.5	47.6	43.2	38.2	0.81	(0.71,0.94)	0.005	0.64	(0.48,0.87)	0.004

Structured diabetes education offered	17.2	22.5	25	48.3	40.2	31.0	1.17	(1.10,1.24)	<0.001	1.44	(1.32,1.56)	<0.001
Risk assessment												
CVD risk score	15.4	20.6	18.6	43.4	34.2	30.8	1.02	(0.96,1.09)	0.463	1.06	(0.96,1.16)	0.249
NHS Health Check	1.7	2.8	3.1	52.1	44.9	38.1	1.30	(1.10,1.54)	0.002	1.32	(1.05,1.67)	0.019

*All models adjust for age at baseline, sex, deprivation, raised HbA1c at baseline, and clustering by practice. Time to initiation of antidiabetic therapy restricted to those free from antidiabetic medication in 12 months prior to diagnosis date.

### 3.4 Sensitivity analysis

Compared to those of known ethnicity (n = 126,331), individuals of unknown ethnicity (n = 53,575) were younger at diagnosis (β = -1.13, 95%CI −1.32, −0.94), had reduced odds of risk factors recording in the 12 months prior to diagnosis for 9/10 measures, and slower initiation of therapeutic and non-therapeutic management post diagnosis compared to those of known ethnicity (p < 0.001). While individuals of unknown ethnicity had poorer control of HbA1c, FBG, and blood pressure, they had more favourable cholesterol, BMI and serum creatinine levels (p < 0.009). Contrary to expectations, individuals of unknown ethnicity had greater odds of being in the most affluent quintile of deprivation relative to those of known ethnicity (OR 1.14, 95%CI 1.07, 1.21), and a lower prevalence of gestational diabetes, vascular disease, and medication use (p < 0.001, appendix figures S2 S3, appendix tables S1,S2).

## 4 Discussion

We report the findings of a large population-based cohort study examining ethnic differences in both the clinical characteristics and clinical management of type two diabetes at time of diagnosis. The results show that, despite a lower consultation rate and higher burden of pre-diabetic states, south Asian and black groups had better capture of risk factors, a lower age at diagnosis, and better or equivalent cardio-metabolic profile at diagnosis. Initiation of antidiabetic treatment was faster for black and south Asian individuals, as was time to first NHS health check and time to offer of structured education. However, time to first consultation and measurement of risk factors was largely slower for non-white groups.

Overall, our findings suggest that downstream inequalities in diabetes outcomes do not appear to stem wholly from inequalities around the time of initial diagnosis, and in fact, highlight several positive aspects of primary care-based diabetes management. Firstly, the similarity of microvascular disease between ethnic groups at time of diagnosis suggests that non-white groups are not being diagnosed at a more severe stage of diabetes progression, and that the latency between true onset of diabetes and clinical diagnosis does not disadvantage ethnic minority groups. Combined with the findings of pro-active treatment initiation and timely risk assessments, our findings suggest that the elevated burden of cardio-metabolic risk in non-white groups is being appropriately recognized by health care professionals. Delays in risk factor measurement and diabetes review may reflect lower burden of cardio-metabolic risk at time of diagnosis or may be indicative of growing ethnic disparities with respect to longer-term diabetes management.

### 4.1 Comparisons with existing literature

To date, only two other UK based studies have reported ethnic differences in clinical severity at initial diagnosis of type 2 diabetes [21], [22]. The first, a London based study of 1506 individuals, found that diagnosis was ten years earlier for both black and south Asian populations, and that both non-white groups had lower levels of glycaemia and vascular complications [21]. The second was a 2003 survey of 1899 individuals with type 2 diabetes which reported equivalent access to diabetes care between black and white individuals- providing further support to our findings of equity between ethnic groups with respect to clinical care before and after diagnosis [22].

Improvements in both quality of diabetes care and risk factor profiles of people with type 2 diabetes in the UK may be related to several overlapping causes. Firstly, the introduction of the Quality and Outcomes Framework (QOF), which incentivises achievement of quality targets for the care of individuals with chronic conditions, has both improved overall standards of diabetes care and reduced variations in diabetes care provision [23], [24], [25]. However, one study found that, though QOF incentivisation had accelerated short-term improvements in blood pressure and cholesterol, ethnic disparities in HbA1c remained – a finding echoed in our own study which showed that black people had significantly higher HbA1c at diagnosis, despite equivalence of other risk factors [26].

Secondly, awareness amongst health care practitioners about ethnic differences in cardio-metabolic risk has increased steadily and may be responsible for the pro-active management of diabetes in non-white groups. Ethnicity has now been incorporated into clinical guidance documents for hypertension, obesity, type 2 diabetes, and smoking cessation [27], [28], [29]. Specifically, guidelines for the prevention of type 2 diabetes in people at high risk, specify that individuals from ethnic minority populations should be encouraged to undergo a risk assessment for type 2 diabetes [20]. In May 2018, a new guideline for promoting health amongst ethnic minority groups, was published – indicating that further reductions in ethnic disparities may become apparent over time [30].

Thirdly, improvements in risk factor profiles at diagnosis may be part of a larger trend of decreasing vascular disease across the developed world [31]. A 2017 study of trends in type 2 diabetes incidence, prevalence and mortality in the UK found a 32% decrease in all-cause mortality between 2004 and 2014, and a 2% increase in prevalence, thought to be driven by better survival rather than increasing incidence [32]. The findings of our study reflect these emerging trends, with reductions in ethnic inequalities likely driven by temporal improvements in population levels of risk factors, quality of clinical care, awareness of established ethnic differences in outcomes, and increased effectiveness of novel pharmacological therapies.

### 4.2 Strengths

The strengths and limitations of routine electronic health records (EHRs) for diabetes research have been comprehensively outlined in a recent review [33]. In this study, the sample size was large and drawn from a representative denominator population, allowing sufficient power to detect differences between the main ethnic groups in the UK. The cohort was identified using a validated algorithm, designed to minimize misclassification of diabetes type [34]. In order to account for the step change in diabetes management following the introduction of QOF, entry into the study cohort was restricted to individuals diagnosed with type 2 diabetes from 2004 onwards. Recent improvements in the completeness of ethnicity recording in the CPRD as part of QOF have facilitated a more robust examination of ethnic differences in conditions managed largely in primary care [17]. Linkage to deprivation data enabled us to separate the influences of ethnicity and deprivation, which are often conflated when examining health disparities. Restricting the study sample to people with at least 6 months of continuous registration prior to their initial diagnosis of type 2 diabetes ensured that diagnoses were truly incident and that all outcomes of interest were measured as close to initiation of diabetes management as possible. General practice characteristics such as size, and participation in local enhanced service schemes have been found to play a large role in observed variations in quality of diabetes care [35]. By accounting for the clustering of people within practices, we were able to appropriately account for the influence of practice level factors on ethnic disparities.

### 4.3 Limitations

As EHRs are primarily used for patient care rather than research, data quality and completeness can vary significantly depending on the time-period, disease area, and indicator of interest. Though financial incentivisation has standardized many aspects of diabetes care, shared decision is now the preferred model for management of many long-term conditions. As such, observed differences in diabetes indicators may be due to active choices by the individual and provider to deviate from standard management plans in order to manage competing priorities. Ethnicity data was not available for 30% of the study cohort, which may have introduced bias. Since ethnicity data are unlikely to be missing at random, it would have been inappropriate to impute these data. Sensitivity analyses showed that individuals with unknown ethnicity were younger at diagnosis and, surprisingly, less deprived than those of known ethnicity. Coupled with the findings of heterogeneity in clinical profile at diagnosis (poorer risk factor levels but fewer co-morbidities), it is likely that this is a mixed group encompassing younger, healthier, and more affluent individuals who may not need to access healthcare, and individuals who are less healthy, or less able to access care. Deprivation scores derived from the postcode of the general practice were used for 40% of participants without permissions for linkage to individual level data. The relationship between practice level and individual level deprivation will vary greatly between individuals, potentially underestimating the true confounding effect of deprivation on the association between ethnicity and diabetes when using practice level as a proxy.

The dataset did not include information on genetic risk factors, early life exposures, migration history, diet and exercise, or any measures of health seeking behaviour or differences in attitudes towards medications and thus unmeasured confounding may have influenced the results. Future studies combining routine EHRs with cohort studies such as the UK Biobank will be valuable in obtaining a complete picture of an individual’s health across the life course.

### 4.4 Conclusions

Overall, we find limited evidence of systematic ethnic inequalities in identification of type 2 diabetes and management of cardio-metabolic risk around the time of initial diagnosis. Findings from this study may be illustrative of a wider trend of shrinking inequalities in diabetes care. Additional investigations into the origin and implications of missingness of ethnicity data are warranted. Future work examining the extent to which ethnic differences are explained by genetic factors and whether ethnic disparities manifest later in the care pathway, for example, in relation to long-term risk factor control as suggested here, will be necessary to understand how patterns of ethnic disparities in risk factor control and long-term outcomes are evolving in the UK.

## Funding

RM is supported by a Sir Henry Wellcome Postdoctoral Fellowship from the Wellcome Trust (201375/Z/16/Z). The study sponsor was not involved in the design of the study; the collection, analysis, and interpretation of data; writing the report; or the decision to submit the report for publication.

## 6 Contribution statement

RM conceived the study, curated the data, conducted the main statistical analysis and authored the initial manuscript. LP conducted statistical analysis and contributed to the manuscript. RF provided statistical advice and contributed to the manuscript. NC and LS helped conceive the study and contributed to the manuscript. Rohini Mathur is the guarantor for this project.

## 7 Data availability

The data used for this study comprises anonymised patient records derived from the CPRD. Only the authors have access to the CPRD data. Code lists are available in the supplementary materials and will be uploaded to the LSHTM data compass (http://datacompass.lshtm.ac.uk). Researchers should contact the CPRD’s Independent Scientific Advisory Committee (ISAC) to obtain access to data.

## Declaration of Competing Interest

The authors declare no conflict of interest.
