# Supplementary material for: Ethnic differences in the severity and clinical management of type 2 diabetes at time of diagnosis: A cohort study in the UK Clinical Practice Research Datalink
Source: Diabetes Res Clin Pract. 2020 Feb;160:108006. doi: 10.1016/j.diabres.2020.108006 (PMC7042884; doi:10.1016/j.diabres.2020.108006)
Supplement: Supplementary data 1 [file mmc1.docx]

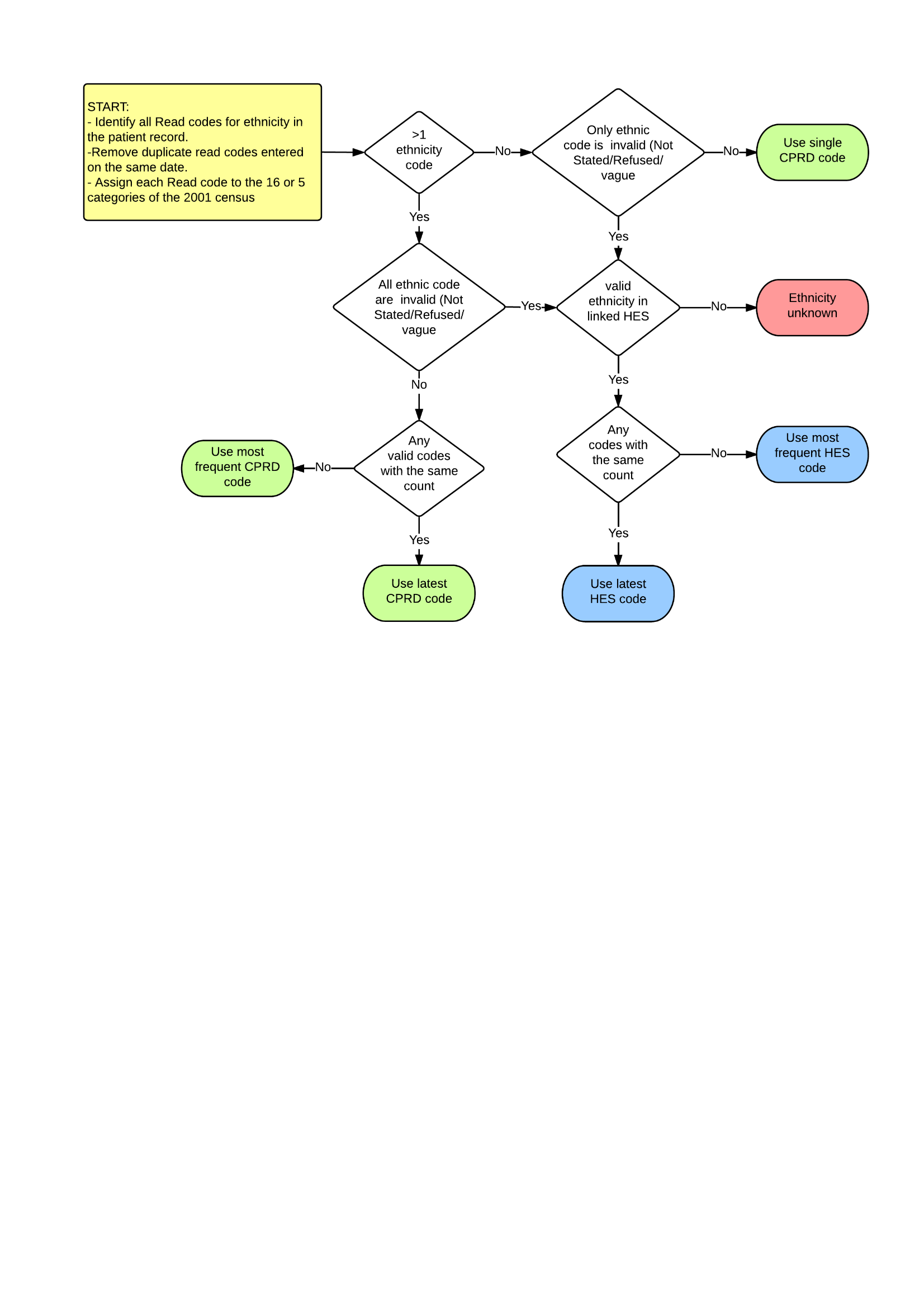


Figure S1. Algorithm to assign ethnicity to study participants (This study had access to CPRD data only)


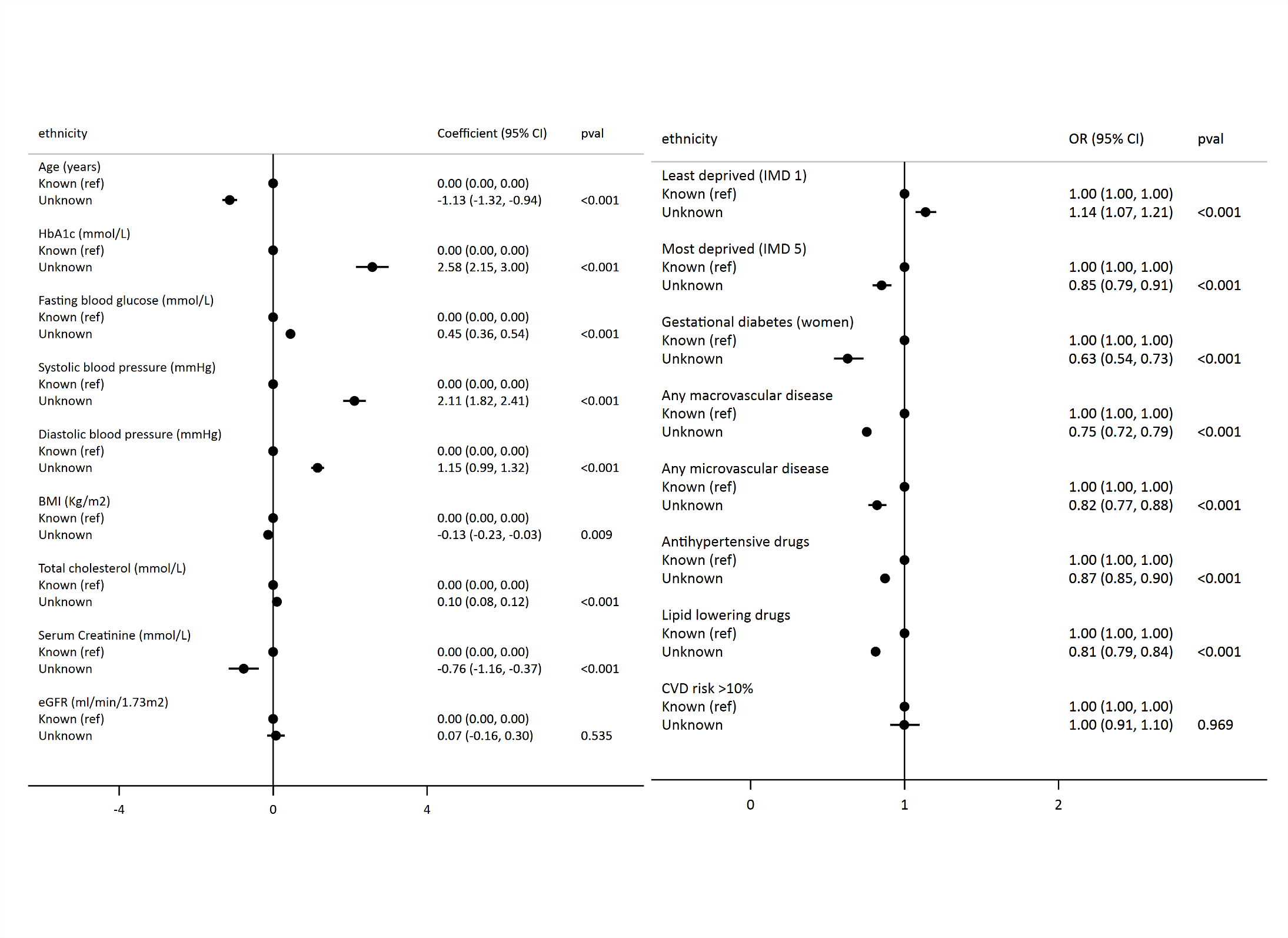


Figure S2: Clinical severity of diabetes at diagnosis for those of known ethnicity compared to those of unknown ethnicity

| Table S1. Risk factor recording in the 12 months prior to T2DM diagnosis: Known vs. Unknown ethnicity | | | | | |
| --- | --- | --- | --- | --- | --- |
|  | % with risk factor recorded | | Adjusted difference | | |
|  | **Known** | **Unknown** | **Unknown vs Known** | | |
| N | 126,311 | 53,575 |  |  |  |
|  | % | % | OR | 95%CI | p.val |
| HbA1c | 52.8 | 53.0 | 0.86 | (0.83,0.88) | <0.001 |
| Glucose | 65.7 | 68.1 | 0.80 | (0.78,0.82) | <0.001 |
| Blood Pressure | 88.6 | 87.0 | 0.76 | (0.74,0.79) | <0.001 |
| BMI | 64.2 | 62.2 | 0.84 | (0.82,0.87) | <0.001 |
| Total Cholesterol | 79.0 | 76.3 | 0.70 | (0.69,0.72) | <0.001 |
| Creatinine | 87.5 | 85.9 | 0.65 | (0.63,0.67) | <0.001 |
| Urine ACR | 2.4 | 2.8 | 0.76 | (0.96,1.44) | 0.117 |
| Smoking Status | 72.7 | 70.9 | 0.72 | (0.69,0.76) | <0.001 |
| Risk assessments |  |  |  |  |  |
| NHS Health Check | 4.6 | 2.0 | 0.81 | (0.74,0.89) | <0.001 |
| CVD risk score | 19.1 | 14.2 | 0.78 | (0.75,0.82) | <0.001 |
| Consultations | Median (IQR) | | β | CI95% | p.val |
| Number of consultations | 10 (6-17) | 9 (5-15) | -3.00 | (-3.14,-2.82) | <0.001 |

Table S2. Time to first clinical management following T2DM diagnosis: Known vs. Unknown ethnicity

|  | **% receiving clinical management** | | **Median time to first clinical event (months)** | | **Adjusted HR** | | |
| --- | --- | --- | --- | --- | --- | --- | --- |
|  |  |  |  |  |  |  |  |
|  | **Known** | **Unknown** | **Known** | **Unknown** | **Unknown vs Known** | | |
|  |  |  |  |  | **HR** | **CI95%** | **p.val** |
| **First post-diagnosis consultation** | 2.6 | 1.5 | 50.5 | 50.4 | 0.96 | (0.87,1.07) | 0.488 |
| **First antidiabetic medication** | 94.5 | 93.9 | 2.8 | 2.7 | 0.95 | (0.93,0.96) | <0.001 |
| **Risk Factor Measurement** |  |  |  |  |  |  |  |
| Fasting blood glucose | 92.2 | 91 | 3.8 | 3.6 | 0.95 | (0.94,0.97) | <0.001 |
| HbA1c | 92.8 | 91.9 | 3.0 | 2.9 | 0.91 | (0.90,0.93) | <0.001 |
| Urine ACR | 73.9 | 72.1 | 3.4 | 3.9 | 0.89 | (0.87,0.91) | <0.001 |
| BMI | 38.7 | 34.7 | 26.7 | 26.0 | 0.95 | (0.93,0.98) | <0.001 |
| Blood Pressure | 6 | 4.8 | 47.0 | 47.6 | 0.99 | (0.92,1.06) | 0.723 |
| Cholesterol | 17.7 | 17.3 | 47.2 | 47.3 | 0.99 | (0.95,1.02) | 0.438 |
| Creatinine | 15.7 | 13.2 | 42.4 | 44.2 | 1.05 | (1.01,1.09) | 0.025 |
| CVD Risk score | 1.8 | .6 | 51.1 | 51.0 | 0.65 | (0.55,0.75) | <0.001 |
|  |  |  |  |  |  |  |  |
| **Diabetes Review** |  |  |  |  |  |  |  |
| Dietary Advice | 99.9 | 99.8 | 0.1 | 0.1 | 0.91 | (0.89,0.92) | <0.001 |
| Diabetes review | 61.3 | 65.3 | 10.4 | 8.0 | 0.92 | (0.90,0.94) | <0.001 |
| Retinopathy screen | 58.6 | 67.1 | 11.4 | 8.0 | 0.90 | (0.88,0.92) | <0.001 |
| Foot examination | 96.1 | 95.7 | 1.4 | 1.4 | 0.93 | (0.91,0.94) | <0.001 |
|  |  |  |  |  |  |  |  |
| **Structured diabetes education offered** | 92.5 | 91.4 | 1.8 | 1.7 | 0.91 | (0.90,0.92) | <0.001 |
|  |  |  |  |  |  |  |  |
| **Risk Assessment** |  |  |  |  |  |  |  |
| CVD risk score | 1.8 | .6 | 51.1 | 51.0 | 0.65 | (0.55,0.75) | <0.001 |
| NHS Health Check | 93.9 | 93.3 | 3.3 | 3.1 | 0.92 | (0.91,0.94) | <0.001 |

*All models adjust for age at baseline, sex, deprivation, raised HbA1c at baseline, and clustering by practice. Time to initiation of antidiabetic therapy restricted to those free from antidiabetic medication in 12 months prior to diagnosis date

Figure S3. Correspondence analysis plot obtained from the contingency table cross-classifying ethnicity and IMD deprivation quintile

Notes: Correspondence analysis allows visualisation of relationships between two categorical variables in a two-dimensional biplot whereby a measure of association of categories of the two variables (ethnicity and deprivation) is given by the inner product of the vectors that link them to the origin. The distance of the profile points of deprivation and ethnicity from the origin defines their distance from the average socio-economic and ethnic profile. CA biplots capturing 99.4% of the dispersion in the data show a strong association between Black, South-Asian, Mixed and Other groups and the 5th quintile of deprivation (their distance from the origin is large/moderate and the angle between them acute/negligible). The white group appears to be associated with the second and third quintiles of deprivation, although less strongly as they are closer to the origin (i.e. the average deprivation profile). The group of unknown ethnicity appears to be associated with both the 4^th^ and 1^st^ quintile of deprivation (moderate distance from the origin and acute angle with both quintiles), suggesting this group has a more heterogenous socio-economic composition.

Table S3. Codelists for all study variables

| CPRD Medcode | Readcode | Readterm |  |  |
| --- | --- | --- | --- | --- |
| Ethnicity |  |  | Ethnicity in 16 categories | Ethnicity in 5 cateories |
| 10196 | 9S...00 | Ethnic groups (1991 census) | Not Stated | Not Stated |
| 22467 | 9S1..00 | White | British | White |
| 12446 | 9S10.00 | White British | British | White |
| 24837 | 9S11.00 | White Irish | Irish | White |
| 12444 | 9S12.00 | Other white ethnic group | Other White | White |
| 26467 | 9S13.00 | White Scottish | Other White | White |
| 26310 | 9S14.00 | Other white British ethnic group | British | White |
| 12632 | 9S2..00 | Black Caribbean | Caribbean | Black |
| 12778 | 9S3..00 | Black African | African | Black |
| 24339 | 9S4..00 | Black, other, non-mixed origin | Other Black | Black |
| 12452 | 9S41.00 | Black British | Other Black | Black |
| 57435 | 9S42.00 | Black Caribbean/W.I./Guyana | Other Black | Black |
| 47950 | 9S42.11 | Black Caribbean | Other Black | Black |
| 47997 | 9S42.12 | Black West Indian | Other Black | Black |
| 32100 | 9S42.13 | Black Guyana | Other Black | Black |
| 41329 | 9S43.00 | Black N African/Arab/Iranian | Other Black | Black |
| 46812 | 9S43.11 | Black North African | Other Black | Black |
| 57752 | 9S43.12 | Black Arab | Other Black | Black |
| 50286 | 9S43.13 | Black Iranian | Other Black | Black |
| 35412 | 9S44.00 | Black - other African country | African | Black |
| 47965 | 9S45.00 | Black E Afric Asia/Indo-Caribb | Other Black | Black |
| 57753 | 9S45.11 | Black East African Asian | Other Black | Black |
| 57763 | 9S45.12 | Black Indo-Caribbean | Other Black | Black |
| 48005 | 9S46.00 | Black Indian sub-continent | Other Black | Black |
| 35350 | 9S47.00 | Black - other Asian | Other Black | Black |
| 26312 | 9S48.00 | Black Black - other | Other Black | Black |
| 25676 | 9S5..00 | Black - other, mixed | Other Black | Black |
| 25623 | 9S51.00 | Other Black - Black/White orig | Other Mixed | Mixed |
| 32165 | 9S52.00 | Other Black - Black/Asian orig | Other Mixed | Mixed |
| 12482 | 9S6..00 | Indian | Indian | South Asian |
| 24690 | 9S7..00 | Pakistani | Pakistani | South Asian |
| 24740 | 9S8..00 | Bangladeshi | Bangladeshi | South Asian |
| 24272 | 9S9..00 | Chinese | Chinese | Other |
| 30280 | 9SA..00 | Other ethnic non-mixed (NMO) | Other ethnic group | Other |
| 32110 | 9SA1.00 | Brit. ethnic minor. spec.(NMO) | Other ethnic group | Other |
| 57764 | 9SA2.00 | Brit. ethnic minor. unsp (NMO) | Other ethnic group | Other |
| 54593 | 9SA3.00 | Caribbean I./W.I./Guyana (NMO) | Other Black | Black |
| 57094 | 9SA3.11 | Caribbean Island (NMO) | Other Black | Black |
| 57075 | 9SA3.12 | West Indian (NMO) | Other Black | Black |
| 93144 | 9SA3.13 | Guyana (NMO) | Other Black | Black |
| 24962 | 9SA4.00 | N African Arab/Iranian (NMO) | Other ethnic group | Other |
| 47285 | 9SA4.11 | North African Arab (NMO) | Other ethnic group | Other |
| 25082 | 9SA4.12 | Iranian (NMO) | Other ethnic group | Other |
| 47969 | 9SA5.00 | Other African countries (NMO) | African | Black |
| 38097 | 9SA6.00 | E Afric Asian/Indo-Carib (NMO) | Other Asian | South Asian |
| 46818 | 9SA6.11 | East African Asian (NMO) | Other Asian | South Asian |
| 99316 | 9SA6.12 | Indo-Caribbean (NMO) | Other Asian | South Asian |
| 39696 | 9SA7.00 | Indian sub-continent (NMO) | Other Asian | South Asian |
| 26379 | 9SA8.00 | Other Asian (NMO) | Other Asian | South Asian |
| 24270 | 9SA9.00 | Irish (NMO) | Irish | White |
| 45947 | 9SAA.00 | Greek/Greek Cypriot (NMO) | Other ethnic group | Other |
| 45955 | 9SAA.11 | Greek (NMO) | Other ethnic group | Other |
| 47949 | 9SAA.12 | Greek Cypriot (NMO) | Other ethnic group | Other |
| 32066 | 9SAB.00 | Turkish/Turkish Cypriot (NMO) | Other ethnic group | Other |
| 32126 | 9SAB.11 | Turkish (NMO) | Other ethnic group | Other |
| 32069 | 9SAB.12 | Turkish Cypriot (NMO) | Other ethnic group | Other |
| 12633 | 9SAC.00 | Other European (NMO) | Other ethnic group | Other |
| 41214 | 9SAD.00 | Other ethnic NEC (NMO) | Other ethnic group | Other |
| 12696 | 9SB..00 | Other ethnic, mixed origin | Other Mixed | Mixed |
| 47401 | 9SB1.00 | Other ethnic, Black/White orig | Other Mixed | Mixed |
| 32401 | 9SB2.00 | Other ethnic, Asian/White orig | White and Asian | Mixed |
| 35459 | 9SB3.00 | Other ethnic, mixed white orig | Other Mixed | Mixed |
| 32420 | 9SB4.00 | Other ethnic, other mixed orig | Other Mixed | Mixed |
| 32425 | 9SB5.00 | Black Caribbean and White | White and Black Caribbean | Mixed |
| 32443 | 9SB6.00 | Black African and White | White and Black African | Mixed |
| 25411 | 9SC..00 | Vietnamese | Other ethnic group | Other |
| 12429 | 9SD..00 | Ethnic group not given - patient refused | Not Stated | Not Stated |
| 24340 | 9SE..00 | Ethnic group not recorded | Not Stated | Not Stated |
| 32136 | 9SG..00 | Other black ethnic group | Other Black | Black |
| 12668 | 9SH..00 | Other Asian ethnic group | Other Asian | South Asian |
| 47601 | 9SI..00 | Irish traveller | Irish | White |
| 12757 | 9SJ..00 | Other ethnic group | Other ethnic group | Other |
| 45199 | 9SZ..00 | Ethnic groups (census) NOS | Not Stated | Not Stated |
| 12435 | 9i...00 | Ethnic category - 2001 census | Not Stated | Not Stated |
| 12351 | 9i0..00 | British or mixed British - ethnic category 2001 census | British | White |
| 98111 | 9i00.00 | White British - ethnic category 2001 census | British | White |
| 12532 | 9i1..00 | Irish - ethnic category 2001 census | Irish | White |
| 98213 | 9i10.00 | White Irish - ethnic category 2001 census | Irish | White |
| 12421 | 9i2..00 | Other White background - ethnic category 2001 census | Other White | White |
| 12352 | 9i20.00 | English - ethnic category 2001 census | Other White | White |
| 12436 | 9i21.00 | Scottish - ethnic category 2001 census | Other White | White |
| 12681 | 9i22.00 | Welsh - ethnic category 2001 census | Other White | White |
| 28887 | 9i23.00 | Cornish - ethnic category 2001 census | Other White | White |
| 42294 | 9i24.00 | Northern Irish - ethnic category 2001 census | Other White | White |
| 40102 | 9i25.00 | Ulster Scots - ethnic category 2001 census | Other White | White |
| 32778 | 9i26.00 | Cypriot (part not stated) - ethnic category 2001 census | Other White | White |
| 12355 | 9i27.00 | Greek - ethnic category 2001 census | Other White | White |
| 12769 | 9i28.00 | Greek Cypriot - ethnic category 2001 census | Other White | White |
| 12746 | 9i29.00 | Turkish - ethnic category 2001 census | Other White | White |
| 32413 | 9i2A.00 | Turkish Cypriot - ethnic category 2001 census | Other White | White |
| 12412 | 9i2B.00 | Italian - ethnic category 2001 census | Other White | White |
| 55223 | 9i2C.00 | Irish Traveller - ethnic category 2001 census | Other White | White |
| 55113 | 9i2D.00 | Traveller - ethnic category 2001 census | Other White | White |
| 42290 | 9i2E.00 | Gypsy/Romany - ethnic category 2001 census | Other White | White |
| 12467 | 9i2F.00 | Polish - ethnic category 2001 census | Other White | White |
| 12433 | 9i2G.00 | Baltic Estonian/Latvian/Lithuanian - ethn categ 2001 census | Other White | White |
| 28973 | 9i2H.00 | Commonwealth (Russian) Indep States - ethn categ 2001 census | Other White | White |
| 26341 | 9i2J.00 | Kosovan - ethnic category 2001 census | Other White | White |
| 25422 | 9i2K.00 | Albanian - ethnic category 2001 census | Other White | White |
| 46956 | 9i2L.00 | Bosnian - ethnic category 2001 census | Other White | White |
| 28866 | 9i2M.00 | Croatian - ethnic category 2001 census | Other White | White |
| 47074 | 9i2N.00 | Serbian - ethnic category 2001 census | Other White | White |
| 28936 | 9i2P.00 | Other republics former Yugoslavia - ethnic categ 2001 census | Other White | White |
| 26391 | 9i2Q.00 | Mixed Irish and other White - ethnic category 2001 census | Other White | White |
| 12402 | 9i2R.00 | Oth White European/European unsp/Mixed European 2001 census | Other White | White |
| 28900 | 9i2S.00 | Other mixed White - ethnic category 2001 census | Other White | White |
| 12591 | 9i2T.00 | Other White or White unspecified ethnic category 2001 census | Other White | White |
| 12742 | 9i3..00 | White and Black Caribbean - ethnic category 2001 census | White and Black Caribbean | Mixed |
| 12437 | 9i4..00 | White and Black African - ethnic category 2001 census | White and Black African | Mixed |
| 12638 | 9i5..00 | White and Asian - ethnic category 2001 census | White and Asian | Mixed |
| 12873 | 9i6..00 | Other Mixed background - ethnic category 2001 census | Other Mixed | Mixed |
| 12795 | 9i60.00 | Black and Asian - ethnic category 2001 census | Other Mixed | Mixed |
| 49940 | 9i61.00 | Black and Chinese - ethnic category 2001 census | Other Mixed | Mixed |
| 40110 | 9i62.00 | Black and White - ethnic category 2001 census | Other Mixed | Mixed |
| 12706 | 9i63.00 | Chinese and White - ethnic category 2001 census | Other Mixed | Mixed |
| 47005 | 9i64.00 | Asian and Chinese - ethnic category 2001 census | Other Mixed | Mixed |
| 32408 | 9i65.00 | Other Mixed or Mixed unspecified ethnic category 2001 census | Other Mixed | Mixed |
| 12414 | 9i7..00 | Indian or British Indian - ethnic category 2001 census | Indian | South Asian |
| 12460 | 9i8..00 | Pakistani or British Pakistani - ethnic category 2001 census | Pakistani | South Asian |
| 28888 | 9i9..00 | Bangladeshi or British Bangladeshi - ethn categ 2001 census | Bangladeshi | South Asian |
| 12513 | 9iA..00 | Other Asian background - ethnic category 2001 census | Other Asian | South Asian |
| 26392 | 9iA1.00 | Punjabi - ethnic category 2001 census | Other Asian | South Asian |
| 64133 | 9iA2.00 | Kashmiri - ethnic category 2001 census | Other Asian | South Asian |
| 47077 | 9iA3.00 | East African Asian - ethnic category 2001 census | Other Asian | South Asian |
| 12608 | 9iA4.00 | Sri Lankan - ethnic category 2001 census | Other Asian | South Asian |
| 12760 | 9iA5.00 | Tamil - ethnic category 2001 census | Other Asian | South Asian |
| 12887 | 9iA6.00 | Sinhalese - ethnic category 2001 census | Other Asian | South Asian |
| 32399 | 9iA7.00 | Caribbean Asian - ethnic category 2001 census | Other Asian | South Asian |
| 12653 | 9iA8.00 | British Asian - ethnic category 2001 census | Other Asian | South Asian |
| 46056 | 9iA9.00 | Mixed Asian - ethnic category 2001 census | Other Asian | South Asian |
| 28935 | 9iAA.00 | Other Asian or Asian unspecified ethnic category 2001 census | Other Asian | South Asian |
| 12432 | 9iB..00 | Caribbean - ethnic category 2001 census | Caribbean | Black |
| 12350 | 9iC..00 | African - ethnic category 2001 census | African | Black |
| 32389 | 9iD..00 | Other Black background - ethnic category 2001 census | Other Black | Black |
| 12443 | 9iD0.00 | Somali - ethnic category 2001 census | Other Black | Black |
| 32886 | 9iD1.00 | Nigerian - ethnic category 2001 census | Other Black | Black |
| 40097 | 9iD2.00 | Black British - ethnic category 2001 census | Other Black | Black |
| 40096 | 9iD3.00 | Mixed Black - ethnic category 2001 census | Other Black | Black |
| 46047 | 9iD4.00 | Other Black or Black unspecified ethnic category 2001 census | Other Black | Black |
| 12468 | 9iE..00 | Chinese - ethnic category 2001 census | Chinese | Other |
| 12434 | 9iF..00 | Other - ethnic category 2001 census | Other ethnic group | Other |
| 12719 | 9iF0.00 | Vietnamese - ethnic category 2001 census | Other ethnic group | Other |
| 12473 | 9iF1.00 | Japanese - ethnic category 2001 census | Other ethnic group | Other |
| 12420 | 9iF2.00 | Filipino - ethnic category 2001 census | Other ethnic group | Other |
| 12730 | 9iF3.00 | Malaysian - ethnic category 2001 census | Other ethnic group | Other |
| 63872 | 9iF4.00 | Buddhist - ethnic category 2001 census | Other ethnic group | Other |
| 56127 | 9iF5.00 | Hindu - ethnic category 2001 census | Other ethnic group | Other |
| 46063 | 9iF6.00 | Jewish - ethnic category 2001 census | Other ethnic group | Other |
| 47091 | 9iF7.00 | Muslim - ethnic category 2001 census | Other ethnic group | Other |
| 49658 | 9iF8.00 | Sikh - ethnic category 2001 census | Other ethnic group | Other |
| 46059 | 9iF9.00 | Arab - ethnic category 2001 census | Other ethnic group | Other |
| 47028 | 9iFA.00 | North African - ethnic category 2001 census | Other ethnic group | Other |
| 28909 | 9iFB.00 | Mid East (excl Israeli, Iranian & Arab) - eth cat 2001 cens | Other ethnic group | Other |
| 46964 | 9iFC.00 | Israeli - ethnic category 2001 census | Other ethnic group | Other |
| 25937 | 9iFD.00 | Iranian - ethnic category 2001 census | Other ethnic group | Other |
| 45964 | 9iFE.00 | Kurdish - ethnic category 2001 census | Other ethnic group | Other |
| 25451 | 9iFF.00 | Moroccan - ethnic category 2001 census | Other ethnic group | Other |
| 26246 | 9iFG.00 | Latin American - ethnic category 2001 census | Other ethnic group | Other |
| 12756 | 9iFH.00 | South and Central American - ethnic category 2001 census | Other ethnic group | Other |
| 32382 | 9iFJ.00 | Mauritian/Seychellois/Maldivian/St Helena eth cat 2001census | Other ethnic group | Other |
| 26455 | 9iFK.00 | Any other group - ethnic category 2001 census | Other ethnic group | Other |
| 12459 | 9iG..00 | Ethnic category not stated - 2001 census | Not Stated | Not Stated |
| Type 2 diabetes |  |  |  |  |
|  | C10FL | Definite T2 codes |  |  |
|  | C10F4 | Definite T2 codes |  |  |
|  | C10F6 | Definite T2 codes |  |  |
|  | C10FQ | Definite T2 codes |  |  |
|  | C10FP | Definite T2 codes |  |  |
|  | C10F9 | Definite T2 codes |  |  |
|  | C10FG | Definite T2 codes |  |  |
|  | C10FH | Definite T2 codes |  |  |
|  | C10FK | Definite T2 codes |  |  |
|  | C10FJ | Definite T2 codes |  |  |
|  | C10FD | Definite T2 codes |  |  |
|  | C10FR | Definite T2 codes |  |  |
|  | C10FA | Definite T2 codes |  |  |
|  | C10FN | Definite T2 codes |  |  |
|  | C10F3 | Definite T2 codes |  |  |
|  | C10F5 | Definite T2 codes |  |  |
|  | C10FC | Definite T2 codes |  |  |
|  | C10F. | Definite T2 codes |  |  |
|  | C10F2 | Definite T2 codes |  |  |
|  | C10F1 | Definite T2 codes |  |  |
|  | C10FM | Definite T2 codes |  |  |
|  | C10F0 | Definite T2 codes |  |  |
|  | C10FB | Definite T2 codes |  |  |
|  | C10FE | Definite T2 codes |  |  |
|  | C10FF | Definite T2 codes |  |  |
|  | C10F7 | Definite T2 codes |  |  |
|  | C1071 | Possible T2 codes |  |  |
|  | C1051 | Possible T2 codes |  |  |
|  | C1041 | Possible T2 codes |  |  |
|  | C1072 | Possible T2 codes |  |  |
|  | C112z | Possible T2 codes |  |  |
|  | C112. | Possible T2 codes |  |  |
|  | L180X | Possible T2 codes |  |  |
|  | C1021 | Possible T2 codes |  |  |
|  | C1031 | Possible T2 codes |  |  |
|  | C1061 | Possible T2 codes |  |  |
|  | C1001 | Possible T2 codes |  |  |
|  | L1806 | Probable T2 codes |  |  |
|  | C1099 | Probable T2 codes |  |  |
|  | C109J | Probable T2 codes |  |  |
|  | C1090 | Probable T2 codes |  |  |
|  | C109E | Probable T2 codes |  |  |
|  | C109G | Probable T2 codes |  |  |
|  | C109. | Probable T2 codes |  |  |
|  | C1097 | Probable T2 codes |  |  |
|  | C109D | Probable T2 codes |  |  |
|  | C1095 | Probable T2 codes |  |  |
|  | C1091 | Probable T2 codes |  |  |
|  | C109H | Probable T2 codes |  |  |
|  | C1096 | Probable T2 codes |  |  |
|  | C109B | Probable T2 codes |  |  |
|  | C1074 | Probable T2 codes |  |  |
|  | C1094 | Probable T2 codes |  |  |
|  | C109C | Probable T2 codes |  |  |
|  | C109K | Probable T2 codes |  |  |
|  | C1093 | Probable T2 codes |  |  |
|  | C10y1 | Probable T2 codes |  |  |
|  | C1092 | Probable T2 codes |  |  |
|  | C10z1 | Probable T2 codes |  |  |
|  | C109F | Probable T2 codes |  |  |
|  | C109A | Probable T2 codes |  |  |
| Diet control |  |  |  |  |
| 7563 | 66A3.00 | Diabetic on diet only |  |  |
| Smoking status |  |  |  |  |
| medcode | readcode | readterm |  |  |
| 33 | 1371.00 | Never smoked tobacco |  |  |
| 54 | 137..00 | Tobacco consumption |  |  |
| 60 | 137L.00 | Current non-smoker |  |  |
| 90 | 137S.00 | Ex smoker |  |  |
| 93 | 137P.00 | Cigarette smoker |  |  |
| 776 | 137K.00 | Stopped smoking |  |  |
| 1822 | 1376.00 | Very heavy smoker - 40+cigs/d |  |  |
| 1823 | 137P.11 | Smoker |  |  |
| 1878 | 1374.00 | Moderate smoker - 10-19 cigs/d |  |  |
| 3568 | 1375.00 | Heavy smoker - 20-39 cigs/day |  |  |
| 10558 | 137R.00 | Current smoker |  |  |
| 11788 | 1371.11 | Non-smoker |  |  |
| 12240 | 137G.00 | Trying to give up smoking |  |  |
| 12878 | 137T.00 | Date ceased smoking |  |  |
| 12941 | 1372.11 | Occasional smoker |  |  |
| 12942 | 137..11 | Smoker - amount smoked |  |  |
| 12943 | 137J.00 | Cigar smoker |  |  |
| 12944 | 1373.00 | Light smoker - 1-9 cigs/day |  |  |
| 12945 | 137M.00 | Rolls own cigarettes |  |  |
| 12946 | 137F.00 | Ex-smoker - amount unknown |  |  |
| 12947 | 137H.00 | Pipe smoker |  |  |
| 12951 | 137Q.11 | Smoking restarted |  |  |
| 12952 | 137Q.00 | Smoking started |  |  |
| 12955 | 1379.00 | Ex-moderate smoker (10-19/day) |  |  |
| 12956 | 137A.00 | Ex-heavy smoker (20-39/day) |  |  |
| 12957 | 1378.00 | Ex-light smoker (1-9/day) |  |  |
| 12958 | 1372.00 | Trivial smoker - < 1 cig/day |  |  |
| 12959 | 137B.00 | Ex-very heavy smoker (40+/day) |  |  |
| 12960 | 137Z.00 | Tobacco consumption NOS |  |  |
| 12961 | 1377.00 | Ex-trivial smoker (<1/day) |  |  |
| 12962 | 137E.00 | Tobacco consumption unknown |  |  |
| 12963 | 137Y.00 | Cigar consumption |  |  |
| 12964 | 137C.00 | Keeps trying to stop smoking |  |  |
| 12965 | 137X.00 | Cigarette consumption |  |  |
| 12966 | 137V.00 | Smoking reduced |  |  |
| 12967 | 137a.00 | Pipe tobacco consumption |  |  |
| 13351 | 137I.00 | Passive smoker |  |  |
| 19488 | 137O.00 | Ex cigar smoker |  |  |
| 23017 | 137U.00 | Not a passive smoker |  |  |
| 26470 | 137N.00 | Ex pipe smoker |  |  |
| 30423 | 137c.00 | Thinking about stopping smoking |  |  |
| 30762 | 137d.00 | Not interested in stopping smoking |  |  |
| 31114 | 137b.00 | Ready to stop smoking |  |  |
| 32973 | 137W.00 | Chews tobacco |  |  |
| 41979 | 137e.00 | Smoking restarted |  |  |
| 46300 | 137g.00 | Cigarette pack-years |  |  |
| 46321 | 137f.00 | Reason for restarting smoking |  |  |
| 46654 | 137D.00 | Admitted tobacco cons untrue ? |  |  |
| 62686 | 137h.00 | Minutes from waking to first tobacco consumption |  |  |
| 97029 | 137k.00 | Refusal to give smoking status |  |  |
| 97210 | 137j.00 | Ex-cigarette smoker |  |  |
| 99838 | 137K000 | Recently stopped smoking |  |  |
| 100495 | 137l.00 | Ex roll-up cigarette smoker |  |  |
| 101069 | 137I000 | Exposed to tobacco smoke at home |  |  |
| 101338 | 137m.00 | Failed attempt to stop smoking |  |  |
| 105501 | 137o.00 | Waterpipe tobacco consumption |  |  |
| 105711 | 137n.00 | Total time smoked |  |  |
| 106891 | 137i.00 | Ex-tobacco chewer |  |  |
| Creatinine (for eGFR) |  |  |  |  |
| 5 | 44J3.00 | Serum creatinine |  |  |
| 14563 | 46W..00 | Urine microalbumin |  |  |
| 3927 | 44J3300 | Serum creatinine raised |  |  |
| 9430 | 4679.00 | Urine dipstick for protein |  |  |
| 13736 | 44JF.00 | Plasma creatinine level |  |  |
| 13735 | 44HG.00 | Serum creatine kinase level |  |  |
| 31277 | 44J3000 | Serum creatinine abnormal |  |  |
| 26903 | 44J3200 | Serum creatinine normal |  |  |
| 35545 | 44J3100 | Serum creatinine low |  |  |
| 62062 | 44JC.00 | Corrected plasma creatinine level |  |  |
| 42345 | 44J3z00 | Serum creatinine NOS |  |  |
| 45096 | 44JD.00 | Corrected serum creatinine level |  |  |
| Alcohol consumption |  |  |  |  |
| medcode | readcode | readterm |  |  |
| 385 | 1362.11 | Drinks rarely |  |  |
| 669 | E250000 | Nondependent alcohol abuse, unspecified |  |  |
| 749 | 1362.12 | Drinks occasionally |  |  |
| 956 | 136J.00 | Social drinker |  |  |
| 967 | 1367 | Stopped drinking alcohol |  |  |
| 1399 | E23..12 | Alcohol problem drinking |  |  |
| 1618 | 1365.00 | Heavy drinker - 7-9u/day |  |  |
| 2689 | 136G.00 | Beer drinker |  |  |
| 3782 | E250.14 | Intoxication - alcohol |  |  |
| 4447 | 1361.12 | Non-drinker alcohol |  |  |
| 7746 | E250.00 | Nondependent alcohol abuse |  |  |
| 8999 | 136P.00 | Heavy drinker |  |  |
| 9169 | R103.00 | [D]Alcohol blood level excessive |  |  |
| 10161 | 2577.11 | O/E - alcoholic breath |  |  |
| 12271 | E250.11 | Drunkenness NOS |  |  |
| 12949 | 1361 | Teetotaller |  |  |
| 12968 | 136H.00 | Drinks beer and spirits |  |  |
| 12969 | 136I.00 | Drinks wine |  |  |
| 12970 | 1361.11 | Non drinker alcohol |  |  |
| 12971 | 136F.00 | Spirit drinker |  |  |
| 12972 | 1363.00 | Light drinker - 1-2u/day |  |  |
| 12974 | E250200 | Nondependent alcohol abuse, episodic |  |  |
| 12975 | 1362.00 | Trivial drinker - <1u/day |  |  |
| 12977 | 1366.00 | Very heavy drinker - >9u/day |  |  |
| 12979 | 136M.00 | Current non drinker |  |  |
| 12980 | 136N.00 | Light drinker |  |  |
| 12982 | 136K.00 | Alcohol intake above recommended sensible limits |  |  |
| 12983 | 136E.00 | Ex-very heavy drinker-(>9u/d) |  |  |
| 12984 | 136Q.00 | Very heavy drinker |  |  |
| 12985 | 136O.00 | Moderate drinker |  |  |
| 16587 | ZV11311 | [V]Problems related to lifestyle alcohol use |  |  |
| 17777 | E250.13 | Inebriety NOS |  |  |
| 19401 | 136R.00 | Binge drinker |  |  |
| 19493 | 136D.00 | Ex-heavy drinker - (7-9u/day) |  |  |
| 19494 | 136S.00 | Hazardous alcohol use |  |  |
| 19495 | 136C.00 | Ex-moderate drinker - (3-6u/d) |  |  |
| 22933 | 136A.00 | Ex-trivial drinker (<1u/day) |  |  |
| 23610 | E250100 | Nondependent alcohol abuse, continuous |  |  |
| 23978 | U81..00 | [X]Evid of alcohol involv determind by level of intoxication |  |  |
| 24735 | 2577 | O/E - breath - alcohol smell |  |  |
| 26471 | 136B.00 | Ex-light drinker - (1-2u/day) |  |  |
| 26472 | 136L.00 | Alcohol intake within recommended sensible limits |  |  |
| 27518 | E250.12 | Hangover (alcohol) |  |  |
| 28150 | E250z00 | Nondependent alcohol abuse NOS |  |  |
| 30695 | 136T.00 | Harmful alcohol use |  |  |
| 31569 | E250300 | Nondependent alcohol abuse in remission |  |  |
| 44783 | 1D19.00 | Pain in lymph nodes after alcohol consumption |  |  |
| 84218 | 13ZY.00 | Disqualified from driving due to excess alcohol |  |  |
| 94670 | 136W.00 | Alcohol misuse |  |  |
| Coronary Heart Disease (QOF Definition) |  |  |  |  |
| 240 | G3...00 | Ischaemic heart disease |  |  |
| 241 | G30..00 | Acute myocardial infarction |  |  |
| 1204 | G30..14 | Heart attack |  |  |
| 1344 | G340.12 | Coronary artery disease |  |  |
| 1414 | G33z300 | Angina on effort |  |  |
| 1430 | G33..00 | Angina pectoris |  |  |
| 1431 | G311.13 | Unstable angina |  |  |
| 1655 | G340.11 | Triple vessel disease of the heart |  |  |
| 1676 | G3z..00 | Ischaemic heart disease NOS |  |  |
| 1677 | G30..15 | MI - acute myocardial infarction |  |  |
| 1678 | G308.00 | Inferior myocardial infarction NOS |  |  |
| 1792 | G3...13 | IHD - Ischaemic heart disease |  |  |
| 2491 | G30..12 | Coronary thrombosis |  |  |
| 3704 | G307.00 | Acute subendocardial infarction |  |  |
| 3999 | G340000 | Single coronary vessel disease |  |  |
| 4017 | G32..00 | Old myocardial infarction |  |  |
| 4656 | G311.11 | Crescendo angina |  |  |
| 5254 | G340100 | Double coronary vessel disease |  |  |
| 5387 | G301.00 | Other specified anterior myocardial infarction |  |  |
| 5413 | G340.00 | Coronary atherosclerosis |  |  |
| 7320 | G343.00 | Ischaemic cardiomyopathy |  |  |
| 7347 | G311100 | Unstable angina |  |  |
| 7696 | G33z200 | Syncope anginosa |  |  |
| 8935 | G302.00 | Acute inferolateral infarction |  |  |
| 9276 | G31y000 | Acute coronary insufficiency |  |  |
| 9413 | G31y.00 | Other acute and subacute ischaemic heart disease |  |  |
| 9507 | G307000 | Acute non-Q wave infarction |  |  |
| 9555 | G33z500 | Post infarct angina |  |  |
| 10562 | G307100 | Acute non-ST segment elevation myocardial infarction |  |  |
| 11983 | G311500 | Acute coronary syndrome |  |  |
| 12139 | G300.00 | Acute anterolateral infarction |  |  |
| 12229 | G30X000 | Acute ST segment elevation myocardial infarction |  |  |
| 12804 | G33z700 | Stable angina |  |  |
| 13566 | G30..11 | Attack - heart |  |  |
| 13571 | G30..16 | Thrombosis - coronary |  |  |
| 14658 | G30z.00 | Acute myocardial infarction NOS |  |  |
| 14897 | G301z00 | Anterior myocardial infarction NOS |  |  |
| 14898 | G305.00 | Lateral myocardial infarction NOS |  |  |
| 15754 | G34z.00 | Other chronic ischaemic heart disease NOS |  |  |
| 16408 | G32..11 | Healed myocardial infarction |  |  |
| 17307 | G311200 | Angina at rest |  |  |
| 17464 | G32..12 | Personal history of myocardial infarction |  |  |
| 17689 | G30..17 | Silent myocardial infarction |  |  |
| 17872 | G301100 | Acute anteroseptal infarction |  |  |
| 18118 | G311400 | Worsening angina |  |  |
| 18125 | G330000 | Nocturnal angina |  |  |
| 18842 | G35..00 | Subsequent myocardial infarction |  |  |
| 18889 | G34z000 | Asymptomatic coronary heart disease |  |  |
| 19655 | G311.14 | Angina at rest |  |  |
| 20095 | G330.00 | Angina decubitus |  |  |
| 20416 | G3...12 | Atherosclerotic heart disease |  |  |
| 21844 | G31y300 | Transient myocardial ischaemia |  |  |
| 22383 | G3y..00 | Other specified ischaemic heart disease |  |  |
| 23078 | G34y100 | Chronic myocardial ischaemia |  |  |
| 23892 | G304.00 | Posterior myocardial infarction NOS |  |  |
| 24540 | G34y000 | Chronic coronary insufficiency |  |  |
| 24783 | G3...11 | Arteriosclerotic heart disease |  |  |
| 25842 | G33z.00 | Angina pectoris NOS |  |  |
| 26863 | G33z600 | New onset angina |  |  |
| 27951 | G31..00 | Other acute and subacute ischaemic heart disease |  |  |
| 27977 | G31yz00 | Other acute and subacute ischaemic heart disease NOS |  |  |
| 28138 | G34..00 | Other chronic ischaemic heart disease |  |  |
| 28554 | G33zz00 | Angina pectoris NOS |  |  |
| 28736 | G30y000 | Acute atrial infarction |  |  |
| 29421 | G344.00 | Silent myocardial ischaemia |  |  |
| 29643 | G303.00 | Acute inferoposterior infarction |  |  |
| 29758 | G30X.00 | Acute transmural myocardial infarction of unspecif site |  |  |
| 29902 | G330z00 | Angina decubitus NOS |  |  |
| 30330 | G309.00 | Acute Q-wave infarct |  |  |
| 30421 | G30..13 | Cardiac rupture following myocardial infarction (MI) |  |  |
| 32272 | G38..00 | Postoperative myocardial infarction |  |  |
| 32450 | G33z400 | Ischaemic chest pain |  |  |
| 32854 | G30B.00 | Acute posterolateral myocardial infarction |  |  |
| 34328 | G311300 | Refractory angina |  |  |
| 34633 | G34y.00 | Other specified chronic ischaemic heart disease |  |  |
| 34803 | G30y.00 | Other acute myocardial infarction |  |  |
| 35713 | G34yz00 | Other specified chronic ischaemic heart disease NOS |  |  |
| 36523 | G311.00 | Preinfarction syndrome |  |  |
| 36609 | G342.00 | Atherosclerotic cardiovascular disease |  |  |
| 38609 | G351.00 | Subsequent myocardial infarction of inferior wall |  |  |
| 39449 | G312.00 | Coronary thrombosis not resulting in myocardial infarction |  |  |
| 39546 | Gyu3000 | [X]Other forms of angina pectoris |  |  |
| 39655 | G311.12 | Impending infarction |  |  |
| 39693 | G31y200 | Subendocardial ischaemia |  |  |
| 40429 | G301000 | Acute anteroapical infarction |  |  |
| 41221 | G30y200 | Acute septal infarction |  |  |
| 41835 | G384.00 | Postoperative subendocardial myocardial infarction |  |  |
| 45809 | G350.00 | Subsequent myocardial infarction of anterior wall |  |  |
| 46017 | G30yz00 | Other acute myocardial infarction NOS |  |  |
| 46112 | G380.00 | Postoperative transmural myocardial infarction anterior wall |  |  |
| 46166 | G35X.00 | Subsequent myocardial infarction of unspecified site |  |  |
| 46276 | G381.00 | Postoperative transmural myocardial infarction inferior wall |  |  |
| 47637 | Gyu3300 | [X]Other forms of chronic ischaemic heart disease |  |  |
| 52517 | Gyu3.00 | [X]Ischaemic heart diseases |  |  |
| 54251 | G311z00 | Preinfarction syndrome NOS |  |  |
| 54535 | G33z100 | Stenocardia |  |  |
| 55137 | G311011 | MI - myocardial infarction aborted |  |  |
| 61072 | G311000 | Myocardial infarction aborted |  |  |
| 62626 | G30y100 | Acute papillary muscle infarction |  |  |
| 63467 | G306.00 | True posterior myocardial infarction |  |  |
| 66388 | G33z000 | Status anginosus |  |  |
| 68357 | G31y100 | Microinfarction of heart |  |  |
| 68401 | Gyu3200 | [X]Other forms of acute ischaemic heart disease |  |  |
| 68748 | G38z.00 | Postoperative myocardial infarction, unspecified |  |  |
| 72562 | G353.00 | Subsequent myocardial infarction of other sites |  |  |
| 96838 | Gyu3400 | [X]Acute transmural myocardial infarction of unspecif site |  |  |
| 99991 | Gyu3600 | [X]Subsequent myocardial infarction of unspecified site |  |  |
| 105479 | G39..00 | Coronary microvascular disease |  |  |
| 106812 | G383.00 | Postoperative transmural myocardial infarction unspec site |  |  |
| Heart Failure (QOF definition) |  |  |  |  |
| 398 | G580.00 | Congestive heart failure |  |  |
| 884 | G581.00 | Left ventricular failure |  |  |
| 2062 | G58..00 | Heart failure |  |  |
| 2906 | G580.11 | Congestive cardiac failure |  |  |
| 13188 | 662G.00 | Hypertensive treatm.changed |  |  |
| 4024 | G58z.00 | Heart failure NOS |  |  |
| 1223 | G58..11 | Cardiac failure |  |  |
| 21826 | 662F.00 | Hypertension treatm. started |  |  |
| 5942 | G581.13 | Impaired left ventricular function |  |  |
| 13189 | 662g.00 | New York Heart Association classification - class II |  |  |
| 12948 | 662H.00 | Hypertension treatm.stopped |  |  |
| 18853 | 662f.00 | New York Heart Association classification - class I |  |  |
| 19066 | 662h.00 | New York Heart Association classification - class III |  |  |
| 5255 | G581000 | Acute left ventricular failure |  |  |
| 32671 | G580100 | Chronic congestive heart failure |  |  |
| 10079 | G580.12 | Right heart failure |  |  |
| 9524 | G580.14 | Biventricular failure |  |  |
| 17278 | G58z.12 | Cardiac failure NOS |  |  |
| 23707 | G580000 | Acute congestive heart failure |  |  |
| 10154 | G580.13 | Right ventricular failure |  |  |
| 27964 | G582.00 | Acute heart failure |  |  |
| 27884 | G580200 | Decompensated cardiac failure |  |  |
| 23481 | G581.11 | Asthma - cardiac |  |  |
| 51214 | 662i.00 | New York Heart Association classification - class IV |  |  |
| 43618 | G581.12 | Pulmonary oedema - acute |  |  |
| 11424 | G580300 | Compensated cardiac failure |  |  |
| 22262 | G1yz100 | Rheumatic left ventricular failure |  |  |
| 12590 | G58z.11 | Weak heart |  |  |
| 101138 | G583.00 | Heart failure with normal ejection fraction |  |  |
| 94870 | G580400 | Congestive heart failure due to valvular disease |  |  |
| 104275 | G584.00 | Right ventricular failure |  |  |
| 101137 | G583.11 | HFNEF - heart failure with normal ejection fraction |  |  |
| 106897 | G583.12 |  |  |  |
| Ischaemic Stroke |  |  |  |  |
| 5363 | G64..11 | CVA - cerebral artery occlusion |  |  |
| 6155 | G64..13 | Stroke due to cerebral arterial occlusion |  |  |
| 33543 | G6X..00 | Cerebrl infarctn due/unspcf occlusn or sten/cerebrl artrs |  |  |
| 53745 | Gyu6400 | [X]Other cerebral infarction |  |  |
| 40758 | G6W..00 | Cereb infarct due unsp occlus/stenos precerebr arteries |  |  |
| 40053 | G671.00 | Generalised ischaemic cerebrovascular disease NOS |  |  |
| 39403 | G683.00 | Sequelae of cerebral infarction |  |  |
| 91627 | Gyu6300 | [X]Cerebrl infarctn due/unspcf occlusn or sten/cerebrl artrs |  |  |
| 94482 | Gyu6G00 | [X]Cereb infarct due unsp occlus/stenos precerebr arteries |  |  |
| 92036 | Gyu6600 | [X]Occlusion and stenosis of other cerebral arteries |  |  |
| 90572 | Gyu6500 | [X]Occlusion and stenosis of other precerebral arteries |  |  |
| CKD stages 3-5 (QOF) |  |  |  |  |
| 2994 | 7L1A100 | Peritoneal dialysis |  |  |
| 2996 | 7L1A200 | Haemodialysis NEC |  |  |
| 2997 | 7B00.00 | Transplantation of kidney |  |  |
| 5504 | 7B00z00 | Transplantation of kidney NOS |  |  |
| 5911 | ZV42000 | [V]Kidney transplanted |  |  |
| 8037 | 7L1B000 | Insertion of ambulatory peritoneal dialysis catheter |  |  |
| 11553 | SP08300 | Kidney transplant failure and rejection |  |  |
| 11745 | 7B00100 | Transplantation of kidney from live donor |  |  |
| 11773 | 7L1A.11 | Dialysis for renal failure |  |  |
| 12479 | 1Z13.00 | Chronic kidney disease stage 4 |  |  |
| 12585 | 1Z14.00 | Chronic kidney disease stage 5 |  |  |
| 18774 | TB00111 | Renal transplant with complication, without blame |  |  |
| 20073 | 7L1A000 | Renal dialysis |  |  |
| 22252 | ZV45100 | [V]Renal dialysis status |  |  |
| 23773 | 7L1B100 | Removal of ambulatory peritoneal dialysis catheter |  |  |
| 24361 | 7B00200 | Transplantation of kidney from cadaver |  |  |
| 26862 | 7B06300 | Exploration of renal transplant |  |  |
| 28158 | TB11.00 | Kidney dialysis with complication, without blame |  |  |
| 30709 | 7L1C000 | Insertion of temporary peritoneal dialysis catheter |  |  |
| 30756 | 7L1A500 | Continuous ambulatory peritoneal dialysis |  |  |
| 36442 | 7L1B.11 | Placement ambulatory dialysis apparatus - compens renal fail |  |  |
| 45160 | ZV56y11 | [V]Aftercare involving peritoneal dialysis |  |  |
| 46145 | ZV56011 | [V]Aftercare involving renal dialysis NOS |  |  |
| 46438 | SP05613 | [X] Peritoneal dialysis associated peritonitis |  |  |
| 48057 | K0B5.00 | Renal tubulo-interstitial disordrs in transplant rejectn |  |  |
| 48121 | 7B01500 | Transplant nephrectomy |  |  |
| 48639 | SP01500 | Mechanical complication of dialysis catheter |  |  |
| 54844 | U612200 | [X]Failure sterile precautions dur kidney dialys/other perf |  |  |
| 54990 | TB00100 | Kidney transplant with complication, without blame |  |  |
| 55151 | 7B00000 | Autotransplant of kidney |  |  |
| 59315 | SP07G00 | Stenosis of arteriovenous dialysis fistula |  |  |
| 60446 | Z919.00 | Care of haemodialysis equipment |  |  |
| 60498 | Z919300 | Reversing haemodialysis lines |  |  |
| medcode | readcode | readterm |  |  |
| 60743 | ZV56.00 | [V]Aftercare involving intermittent dialysis |  |  |
| 63038 | ZV56z00 | [V]Unspecified aftercare involving intermittent dialysis |  |  |
| 63488 | ZV56y00 | [V]Other specified aftercare involving intermittent dialysis |  |  |
| 63502 | Z91A.00 | Peritoneal dialysis bag procedure |  |  |
| 64828 | 7L1A600 | Peritoneal dialysis NEC |  |  |
| 66705 | 7B00111 | Allotransplantation of kidney from live donor |  |  |
| 66714 | TB11.11 | Renal dialysis with complication, without blame |  |  |
| 69266 | TA22000 | Failure of sterile precautions during kidney dialysis |  |  |
| 69760 | ZVu3G00 | [X]Other dialysis |  |  |
| 70874 | 7B00y00 | Other specified transplantation of kidney |  |  |
| 72004 | 7B01511 | Excision of rejected transplanted kidney |  |  |
| 72336 | Z919100 | Priming haemodialysis lines |  |  |
| 88597 | 7L1A400 | Automated peritoneal dialysis |  |  |
| 89924 | 7B00300 | Allotransplantation of kidney from cadaver, heart-beating |  |  |
| 93366 | 7B0F.00 | Interventions associated with transplantation of kidney |  |  |
| 95122 | 1Z1H.00 | Chronic kidney disease stage 4 with proteinuria |  |  |
| 95405 | 1Z1L.00 | Chronic kidney disease stage 5 without proteinuria |  |  |
| 95406 | 1Z1J.00 | Chronic kidney disease stage 4 without proteinuria |  |  |
| 95508 | 1Z1K.00 | Chronic kidney disease stage 5 with proteinuria |  |  |
| 96133 | 7B00400 | Allotransplantation kidney from cadaver, heart non-beating |  |  |
| 96184 | TA02000 | Accid cut,puncture,perf,h'ge - kidney dialysis |  |  |
| 96347 | 7A61900 | Ligation of arteriovenous dialysis fistula |  |  |
| 97587 | 1Z1J.11 | CKD stage 4 without proteinuria |  |  |
| 97683 | 1Z1L.11 | CKD stage 5 without proteinuria |  |  |
| 98364 | 7B00211 | Allotransplantation of kidney from cadaver |  |  |
| 99160 | 1Z1K.11 | CKD stage 5 with proteinuria |  |  |
| 99312 | 1Z1H.11 | CKD stage 4 with proteinuria |  |  |
| 104963 | K054.00 | Chronic kidney disease stage 4 |  |  |
| 105151 | K055.00 | Chronic kidney disease stage 5 |  |  |
| Diabetic retinopathy |  |  |  |  |
| 1323 | F420.00 | Diabetic retinopathy |  |  |
| 1411 | 3128100 | Fundoscopy abnormal |  |  |
| 1438 | F421000 | Unspecified background retinopathy |  |  |
| 2254 | F424100 | Central serous retinopathy |  |  |
| 2986 | F420200 | Preproliferative diabetic retinopathy |  |  |
| 3286 | F420100 | Proliferative diabetic retinopathy |  |  |
| 3822 | 2BB8.00 | O/E - vitreous haemorrhages |  |  |
| 3837 | F420400 | Diabetic maculopathy |  |  |
| 3914 | 2BB9.00 | O/E - retinal pigmentation |  |  |
| 4514 | 7270011 | Anterior vitrectomy |  |  |
| 6509 | C108700 | Insulin dependent diabetes mellitus with retinopathy |  |  |
| 6702 | F421300 | Hypertensive retinopathy |  |  |
| 6836 | 7271100 | Laser photocoagulation of retina for detachment |  |  |
| 7069 | F420000 | Background diabetic retinopathy |  |  |
| 7890 | F422.00 | Other proliferative retinopathy |  |  |
| 8595 | F42y600 | Retinal exudate or deposit |  |  |
| 8742 | 2BB5.00 | O/E - retinal haemorrhages |  |  |
| 9318 | 7272300 | Laser destruction of lesion of retina |  |  |
| 9339 | F421.00 | Other background retinopathy |  |  |
| 9835 | 2BBL.00 | O/E - diabetic maculopathy present both eyes |  |  |
| 10099 | F420300 | Advanced diabetic maculopathy |  |  |
| 10755 | F420600 | Non proliferative diabetic retinopathy |  |  |
| 10882 | F421400 | Exudative retinopathy |  |  |
| 11053 | F421800 | Retinal microaneurysms NOS |  |  |
| 11129 | 2BBQ.00 | O/E - left eye background diabetic retinopathy |  |  |
| 11433 | 2BBP.00 | O/E - right eye background diabetic retinopathy |  |  |
| 11626 | F420z00 | Diabetic retinopathy NOS |  |  |
| 11858 | 7270400 | Pars plana vitrectomy |  |  |
| 11874 | F422100 | Proliferative retinopathy due to sickle cell disease |  |  |
| 11912 | 5B4..11 | Retinal laser therapy |  |  |
| 13097 | 2BBT.00 | O/E - right eye proliferative diabetic retinopathy |  |  |
| 13099 | 2BBR.00 | O/E - right eye preproliferative diabetic retinopathy |  |  |
| 13101 | 2BBV.00 | O/E - left eye proliferative diabetic retinopathy |  |  |
| 13102 | 2BBW.00 | O/E - right eye diabetic maculopathy |  |  |
| 13103 | 2BBS.00 | O/E - left eye preproliferative diabetic retinopathy |  |  |
| 13106 | 2BB6.00 | O/E - retinal exudates |  |  |
| 13107 | 2BBn.00 | O/E - left eye clinically significant macular oedema |  |  |
| 13108 | 2BBX.00 | O/E - left eye diabetic maculopathy |  |  |
| 17262 | C109600 | Non-insulin-dependent diabetes mellitus with retinopathy |  |  |
| 17293 | 727..00 | Retina and other parts of eye operations |  |  |
| 17916 | F422011 | Retinopathy of prematurity |  |  |
| 18387 | C10E700 | Type 1 diabetes mellitus with retinopathy |  |  |
| 18496 | C10F600 | Type 2 diabetes mellitus with retinopathy |  |  |
| 18775 | 2BBO.00 | O/E - Laser photocoagulation scars |  |  |
| 19532 | 2BB4.00 | O/E - retinal microaneurysms |  |  |
| 19533 | 2BBY.00 | O/E - referable retinopathy |  |  |
| 22871 | C10EP00 | Type 1 diabetes mellitus with exudative maculopathy |  |  |
| medcode | readcode | readterm |  |  |
| 22967 | 2BBF.00 | Retinal abnormality - diabetes related |  |  |
| 25591 | C10FQ00 | Type 2 diabetes mellitus with exudative maculopathy |  |  |
| 25888 | 2BBm.00 | O/E - right eye clinically significant macular oedema |  |  |
| 27022 | 5B42.00 | Laser therapy - retinal lesion |  |  |
| 30477 | F420700 | High risk proliferative diabetic retinopathy |  |  |
| 31829 | F433100 | Solar retinopathy |  |  |
| 34455 | F421112 | Atheroscleritic retinopathy |  |  |
| 35659 | 2BB7.00 | O/E - retinal vascular prolif. |  |  |
| 36035 | F422y00 | Other specified other proliferative retinopathy |  |  |
| 36119 | F421111 | Arterosclerotic retinopathy |  |  |
| 36855 | 2BBG.00 | Retinal abnormality - non-diabetes |  |  |
| 36867 | 2BBa.00 | O/E- non-referable retinopathy |  |  |
| 38096 | F422z00 | Proliferative retinopathy NOS |  |  |
| 38161 | C108711 | Type I diabetes mellitus with retinopathy |  |  |
| 39457 | F421C00 | Other intraretinal microvascular abnormality |  |  |
| 40982 | F421z00 | Other background retinopathy NOS |  |  |
| 41049 | C108712 | Type 1 diabetes mellitus with retinopathy |  |  |
| 41229 | F421100 | Atherosclerotic retinopathy |  |  |
| 42762 | C109612 | Type 2 diabetes mellitus with retinopathy |  |  |
| 45145 | 2BB2.00 | O/E - retinal vessel narrowing |  |  |
| 45876 | F421200 | Renal retinopathy |  |  |
| 46068 | 7272500 | Panretinal laser photocoagulation to lesion of retina NEC |  |  |
| 47328 | 2BBk.00 | O/E - right eye stable treated prolif diabetic retinopathy |  |  |
| 48751 | 2BB3.00 | O/E - retinal A-V nipping |  |  |
| 49655 | C10F611 | Type II diabetes mellitus with retinopathy |  |  |
| 50656 | 2BBc.00 | O/E - No retinal laser photocoagulation scars |  |  |
| 52041 | 2BBl.00 | O/E - left eye stable treated prolif diabetic retinopathy |  |  |
| 52630 | 2BBo.00 | O/E - sight threatening diabetic retinopathy |  |  |
| 55026 | 7270B11 | Anterior vitrectomy |  |  |
| 58604 | C109611 | Type II diabetes mellitus with retinopathy |  |  |
| 65463 | F420800 | High risk non proliferative diabetic retinopathy |  |  |
| 66964 | F426500 | Pseudoretinitis pigmentosa |  |  |
| 69662 | F421G00 | Venostasis retinopathy |  |  |
| 72424 | 7270B00 | Vitrectomy using anterior approach |  |  |
| 86068 | 7272800 | Panretinal laser photocoagulation to lesion of retina |  |  |
| 88368 | 7270411 | Vitrectomy using pars plana approach |  |  |
| 93875 | C10E712 | Insulin dependent diabetes mellitus with retinopathy |  |  |
| 95343 | C10E711 | Type I diabetes mellitus with retinopathy |  |  |
| 96926 | FyuF700 | [X]Other proliferative retinopathy |  |  |
| 97894 | C10EP11 | Type I diabetes mellitus with exudative maculopathy |  |  |
| 100979 | 7272900 | Focal laser photocoagulation of retina |  |  |
| 101881 | 2BBr.00 | Impaired vision due to diabetic retinopathy |  |  |
| 102242 | 2BBs.00 | Retinal arteries silverwire |  |  |
| Neuropathy |  |  |  |  |
| 2342 | F372.12 | Diabetic neuropathy |  |  |
| 2790 | F367.00 | Peripheral neuropathy |  |  |
| 2925 | F375.00 | Alcoholic polyneuropathy |  |  |
| 3958 | F366.00 | Polyneuropathy |  |  |
| 5002 | F372.11 | Diabetic polyneuropathy |  |  |
| 6908 | F36yz00 | Other idiopathic peripheral neuropathy NOS |  |  |
| 7635 | F362.00 | Hereditary sensory neuropathy |  |  |
| 7795 | C106.12 | Diabetes mellitus with neuropathy |  |  |
| 8591 | F35z.11 | Peripheral neuropathy - hereditary or idiopathic |  |  |
| 9193 | F336.00 | Phantom limb syndrome |  |  |
| 10722 | F37..00 | Inflammatory and toxic neuropathy |  |  |
| 11544 | N242300 | Neuropathic pain |  |  |
| 11663 | M271100 | Neuropathic diabetic ulcer - foot |  |  |
| 14883 | F36z.00 | Hereditary or idiopathic peripheral neuropathy NOS |  |  |
| 14884 | F36y.00 | Other idiopathic peripheral neuropathy |  |  |
| 15481 | F37z.00 | Toxic or inflammatory neuropathy NOS |  |  |
| 16230 | C106.00 | Diabetes mellitus with neurological manifestation |  |  |
| 16491 | C106.13 | Diabetes mellitus with polyneuropathy |  |  |
| 18016 | F336000 | Phantom limb syndrome with pain |  |  |
| 18075 | F36..00 | Hereditary and idiopathic peripheral neuropathy |  |  |
| 18425 | C10FB00 | Type 2 diabetes mellitus with polyneuropathy |  |  |
| 18534 | F342400 | Ulnar neuropathy |  |  |
| 19454 | F374A00 | Polyneuropathy in uraemia |  |  |
| 22573 | C106z00 | Diabetes mellitus NOS with neurological manifestation |  |  |
| 24121 | F378.00 | Intercostal neuropathy |  |  |
| 24216 | F370100 | Postinfectious polyneuritis |  |  |
| 24222 | F376.00 | Polyneuropathy due to drugs |  |  |
| 24226 | F37z.11 | Polyneuropathy unspecified |  |  |
| 24355 | F374200 | Polyneuropathy in vitamin B deficiency |  |  |
| 24571 | F372200 | Asymptomatic diabetic neuropathy |  |  |
| 24694 | C108B00 | Insulin dependent diabetes mellitus with mononeuropathy |  |  |
| 28333 | C373200 | Familial neuropathic amyloid |  |  |
| 30537 | F373.00 | Polyneuropathy in malignant disease |  |  |
| 31551 | F37X.00 | Inflammatory polyneuropathy, unspecified |  |  |
| 31790 | F372.00 | Polyneuropathy in diabetes |  |  |
| 32527 | F368.00 | Hereditary motor and sensory neuropathy |  |  |
| 34268 | C10F200 | Type 2 diabetes mellitus with neurological complications |  |  |
| 35465 | F368100 | Hereditary motor and sensory neuropathy type II |  |  |
| 35537 | Fyu7C00 | [X] Polyneuropathy, unspecified |  |  |
| 35785 | F372100 | Chronic painful diabetic neuropathy |  |  |
| 36643 | N035.12 | Neuropathic arthritis |  |  |
| 37315 | F3y0.00 | Diabetic mononeuropathy |  |  |
| 38401 | F360z00 | Hereditary peripheral neuropathy NOS |  |  |
| 39317 | C106100 | Diabetes mellitus, adult onset, + neurological manifestation |  |  |
| 39528 | F360.00 | Hereditary peripheral neuropathy |  |  |
| 39858 | Fyu7B00 | [X]Inflammatory polyneuropathy, unspecified |  |  |
| 40751 | F374900 | Polyneuropathy in sarcoidosis |  |  |
| 41652 | F37y.00 | Other toxic or inflammatory neuropathy |  |  |
| 41716 | C108C00 | Insulin dependent diabetes mellitus with polyneuropathy |  |  |
| 42831 | C10E200 | Type 1 diabetes mellitus with neurological complications |  |  |
| 44095 | F371000 | Polyneuropathy in disseminated lupus erythematosus |  |  |
| 44512 | F364.00 | Idiopathic progressive polyneuropathy |  |  |
| 45081 | F37..11 | Toxic neuropathy |  |  |
| 45467 | C109B00 | Non-insulin dependent diabetes mellitus with polyneuropathy |  |  |
| 45919 | C109212 | Type 2 diabetes mellitus with neurological complications |  |  |
| 46301 | C10EC00 | Type 1 diabetes mellitus with polyneuropathy |  |  |
| 46937 | F365.00 | Neuropathy in association with hereditary ataxia |  |  |
| 47409 | C109B11 | Type II diabetes mellitus with polyneuropathy |  |  |
| 47465 | F371100 | Polyneuropathy in polyarteritis nodosa |  |  |
| 49146 | C108211 | Type I diabetes mellitus with neurological complications |  |  |
| 50527 | C10FB11 | Type II diabetes mellitus with polyneuropathy |  |  |
| 50813 | C109A11 | Type II diabetes mellitus with mononeuropathy |  |  |
| 52089 | F374300 | Polyneuropathy in diphtheria |  |  |
| 52283 | C108200 | Insulin-dependent diabetes mellitus with neurological comps |  |  |
| 54124 | F377.00 | Other toxic agent polyneuropathy |  |  |
| 55076 | Fyu7.00 | [X]Polyneuropathies & other disord of peripheral nerv syst |  |  |
| 55842 | C109200 | Non-insulin-dependent diabetes mellitus with neuro comps |  |  |
| 56159 | Z6P2100 | Control of phantom sensation technique |  |  |
| 56272 | F374.00 | Polyneuropathy in disease EC |  |  |
| 56910 | F368000 | Hereditary motor and sensory neuropathy type I |  |  |
| 57313 | F371.00 | Polyneuropathy in collagen vascular disease |  |  |
| 58758 | F374800 | Polyneuropathy in porphyria |  |  |
| 59903 | C106.11 | Diabetic amyotrophy |  |  |
| medcode | readcode | readterm |  |  |
| 61523 | C106y00 | Other specified diabetes mellitus with neurological comps |  |  |
| 61829 | C108212 | Type 1 diabetes mellitus with neurological complications |  |  |
| 62401 | F371200 | Polyneuropathy in rheumatoid arthritis |  |  |
| 62674 | C10FA00 | Type 2 diabetes mellitus with mononeuropathy |  |  |
| 63555 | F374z00 | Polyneuropathy in disease NOS |  |  |
| 66336 | F374000 | Polyneuropathy in amyloidosis |  |  |
| 67853 | C106000 | Diabetes mellitus, juvenile, + neurological manifestation |  |  |
| 67905 | C109211 | Type II diabetes mellitus with neurological complications |  |  |
| 68105 | C10EB00 | Type 1 diabetes mellitus with mononeuropathy |  |  |
| 68960 | F374500 | Polyneuropathy in hypoglycaemia |  |  |
| 69047 | F37y000 | Serum neuropathy |  |  |
| 71258 | F371z00 | Polyneuropathy in collagen vascular disease NOS |  |  |
| 72320 | C109A00 | Non-insulin dependent diabetes mellitus with mononeuropathy |  |  |
| 72922 | Fyu6B00 | [X]Other mononeuropathies of lower limb |  |  |
| 73337 | F374100 | Polyneuropathy in beriberi |  |  |
| 91741 | Fyu6C00 | [X]Other specified mononeuropathies |  |  |
| 91943 | C10EC11 | Type I diabetes mellitus with polyneuropathy |  |  |
| 93228 | Fyu1300 | [X]Paraneoplastic neuromyopathy and neuropathy |  |  |
| 93868 | Fyu6A00 | [X]Other mononeuropathies of upper limb |  |  |
| 95351 | C10FA11 | Type II diabetes mellitus with mononeuropathy |  |  |
| 96256 | F37y100 | Axonal sensorimotor neuropathy |  |  |
| 97306 | Fyu7200 | [X]Other specified polyneuropathies |  |  |
| 97449 | Fyu7000 | [X]Other hereditary and idiopathic neuropathies |  |  |
| 97479 | Fyu7100 | [X]Other inflammatory polyneuropathies |  |  |
| 97848 | A72x100 | Mumps polyneuropathy |  |  |
| 98616 | C10F211 | Type II diabetes mellitus with neurological complications |  |  |
| 99231 | C108B11 | Type I diabetes mellitus with mononeuropathy |  |  |
| 99855 | M271700 | Neuropathic foot ulcer |  |  |
| 100064 | F374600 | Polyneuropathy in mumps |  |  |
| 101311 | C10EC12 | Insulin dependent diabetes mellitus with polyneuropathy |  |  |
| 101735 | C10E212 | Insulin-dependent diabetes mellitus with neurological comps |  |  |
| 105825 | C373K13 | Familial amyloid polyneuropathy type III |  |  |
| 106103 | F368200 | Hereditary motor and sensory neuropathy type III |  |  |
| 107322 | Fyu6D00 | [X]Other mononeuropathies in diseases classified elsewhere |  |  |
| Hypertension (QOF) |  |  |  |  |
| 204 | G2...00 | Hypertensive disease |  |  |
| 799 | G20..00 | Essential hypertension |  |  |
| 351 | G20..11 | High blood pressure |  |  |
| 15377 | G200.00 | Malignant essential hypertension |  |  |
| 1894 | G201.00 | Benign essential hypertension |  |  |
| 4372 | G202.00 | Systolic hypertension |  |  |
| 83473 | G203.00 | Diastolic hypertension |  |  |
| 10818 | G20z.00 | Essential hypertension NOS |  |  |
| 3712 | G20z.11 | Hypertension NOS |  |  |
| 7329 | G24..00 | Secondary hypertension |  |  |
| 31755 | G240.00 | Secondary malignant hypertension |  |  |
| 73293 | G240z00 | Secondary malignant hypertension NOS |  |  |
| 57288 | G241.00 | Secondary benign hypertension |  |  |
| 51635 | G241z00 | Secondary benign hypertension NOS |  |  |
| 34744 | G244.00 | Hypertension secondary to endocrine disorders |  |  |
| 16059 | G24z.00 | Secondary hypertension NOS |  |  |
| 31387 | G24z000 | Secondary renovascular hypertension NOS |  |  |
| 42229 | G24zz00 | Secondary hypertension NOS |  |  |
| 69753 | Gyu2.00 | [X]Hypertensive diseases |  |  |
| 102458 | Gyu2000 | [X]Other secondary hypertension |  |  |
| Pre-diabetes |  |  |  |  |
| 10921 | C11y200 | Impaired glucose tolerance |  |  |
| 10983 | C11y300 | Impaired fasting glycaemia |  |  |
| 106604 | C11y500 | Pre-diabetes |  |  |
| 10042 | R10E.00 | [D]Impaired glucose tolerance |  |  |
| 10791 | R10D000 | [D]Impaired fasting glycaemia |  |  |
| 31161 | R10D011 | [D]Impaired fasting glucose |  |  |
| 11149 | R102.11 | [D]Prediabetes |  |  |
| 11818 | R102.00 | [D]Glucose tolerance test abnormal |  |  |
| Gestational diabetes |  |  |  |  |
| 10278 | L180800 | Diabetes mellitus arising in pregnancy |  |  |
| 8446 | L180811 | Gestational diabetes mellitus |  |  |
| 2664 | L180900 | Gestational diabetes mellitus |  |  |
| Family history of diabetes |  |  |  |  |
| 23005 | 1253 | FH: Diabetes mellitus in first degree relative |  |  |
| 6795 | 1252 | FH: Diabetes mellitus |  |  |
| Family history of CVD |  |  |  |  |
| 2973 | ZV17312 | [V]Family history of myocardial infarction |  |  |
| 3198 | 12C2.00 | FH: Ischaemic heart dis. <60 |  |  |
| 5970 | ZV17311 | [V]Family history of ischaemic heart disease (IHD) |  |  |
| 6323 | 12C4.12 | FH: Stroke |  |  |
| 6324 | 12C5.00 | FH: Myocardial infarction |  |  |
| 6784 | 12C..13 | FH: Heart disorder |  |  |
| 7207 | 12C3.13 | FH: Angina > 60yrs |  |  |
| 7765 | 12C4.11 | FH: CVA |  |  |
| 8223 | 12C3.00 | FH: Ischaemic heart dis. >60 |  |  |
| 8258 | 12C4.00 | FH: CVA/stroke |  |  |
| 9398 | 1225.00 | No FH: Stroke/TIA |  |  |
| 9490 | 12C5.12 | FH: Ischaemic heart disease |  |  |
| 9528 | 1226.11 | No FH: Angina |  |  |
| 9576 | 12C..11 | FH: CVS disorder |  |  |
| 10934 | 12C5.11 | FH: Coronary thrombosis |  |  |
| 11135 | ZV17300 | [V]Family history of ischaemic heart disease |  |  |
| 11799 | 1226.00 | No FH: Ischaemic heart disease |  |  |
| 12089 | 12C3.12 | FH: MI- myocardial infarct >60 |  |  |
| 12572 | 1225.11 | No FH: CVA/Stroke/TIA |  |  |
| 12709 | 12C2.13 | FH: Angina < 60yrs |  |  |
| 12806 | 12C2.12 | FH: MI- Myocardial infarct <60 |  |  |
| 13222 | 12C..14 | FH: Angina |  |  |
| 13249 | 12C..12 | FH: Cardiac disorder |  |  |
| 13253 | ZV17100 | [V]Family history of stroke (cerebrovascular) |  |  |
| 13258 | 12C..00 | FH: Cardiovascular disease |  |  |
| 13261 | 12C8.00 | FH: Congenital heart disease |  |  |
| 13269 | 12CA.00 | FH myocardial infarction male first degree age known |  |  |
| 13270 | 12C2.11 | FH: Myocardial infarction < 60 |  |  |
| 13274 | 12CJ.00 | FH: Cardiomyopathy |  |  |
| 13275 | 12CQ.00 | Family history of deep vein thrombosis |  |  |
| 18661 | 12CP.00 | FH: Myocardial infarct in 1st degree male relative <55 years |  |  |
| 18997 | 12C7.00 | Family history of transient ischaemic attack |  |  |
| 19127 | 12CN.00 | FH: Myocardial infarct in 1st degree female relative <65 yrs |  |  |
| 19560 | 12C3.11 | FH: Myocardial infarction > 60 |  |  |
| 19561 | 12C7.11 | FH: TIA |  |  |
| 19566 | 1224.00 | No FH: Cardiovascular disease |  |  |
| 23000 | 12CZ.00 | FH: CVS disease NOS |  |  |
| 26625 | 12J3.11 | FH: Congenital heart disease |  |  |
| 26636 | 12CE.00 | FH angina male first degree age known |  |  |
| 26637 | 12CC.00 | FH myocardial infarction female first degree age known |  |  |
| 26639 | 12CK.00 | FH: Aortic aneurysm |  |  |
| 26653 | 12CM.00 | FH: Angina in 1st degree male relative <55 years |  |  |
| 28347 | 12CI.00 | FH: premature coronary heart disease |  |  |
| 29064 | ZV17111 | [V]Family history of cerebrovascular accident (CVA) |  |  |
| 30256 | ZV17400 | [V]Family history of other cardiovascular disease |  |  |
| 30789 | 12CL.00 | FH: Angina in 1st degree female relative <65 years |  |  |
| 34500 | 12J3.00 | FH: Congenital CVS anomaly |  |  |
| 39572 | 12CB.00 | FH myocardial infarction male first degree age unknown |  |  |
| 40865 | 12CF.00 | FH angina male first degree age unknown |  |  |
| 42996 | 12CH.00 | FH angina female first degree age unknown |  |  |
| 43954 | 12CD.00 | FH myocardial infarction female first degree age unknown |  |  |
| 52870 | ZVu6600 | [X]Family hist/ischaemic hrt disease+oth dis/circultr system |  |  |
| 88482 | 12CS.00 | FH: Anomalous coronary artery |  |  |
| 96212 | 12CV.00 | FH: Cardiovascular disease 1st degree male relative < 55 yrs |  |  |
| 96596 | 12CW.00 | FH: Cardiovascular disease 1st degree female reltve < 65 yrs |  |  |
| 103601 | 12CX.00 | Family history of thromboembolic disorder |  |  |
| 106585 | 12CY.00 | FH: peripheral vascular disease |  |  |
| 107341 | 12Ca.00 | FH: Long QT syndrome |  |  |
| CVD Risk Score |  |  |  |  |
| 7913 |  | Coronary heart disease risk |  |  |
| 10128 |  | Cardiovascular event risk |  |  |
| 10302 |  | Framingham coronary heart disease 10 year risk score |  |  |
| 13283 |  | Coronary heart disease risk |  |  |
| 18581 |  | Low risk of primary heart disease |  |  |
| 18948 |  | Moderate risk of primary heart disease |  |  |
| 22210 |  | High risk of primary heart disease |  |  |
| 24721 |  | Framingham coronary heart disease 10 year risk score |  |  |
| 26627 |  | At risk of heart disease |  |  |
| 29433 |  | High risk of heart disease |  |  |
| 36908 |  | UKPDS 10yr coronary heart disease risk score |  |  |
| 43934 |  | Joint British Societies cardiac risk score |  |  |
| 43938 |  | Framingham coronary heart disease 10 yr adjusted risk score |  |  |
| 55103 |  | JBS cardiovascular disease risk 10-20% over next 10 years |  |  |
| 55104 |  | JBS cardiovascular disease risk <10% over next 10 years |  |  |
| 55105 |  | JBS cardiovascular disease risk >30% over next 10 years |  |  |
| 55109 |  | JBS cardiovascular disease risk >20% up to 30% ov next 10 yr |  |  |
| 71748 |  | Coronary heart disease risk clinical management plan |  |  |
| 85854 |  | Review of patient at risk from coronary heart disease |  |  |
| 95889 |  | Assessing cardiovascular risk using SIGN score |  |  |
| QDiabetes Risk score |  |  |  |  |
| 106622 | 38Gj.00 | QDiabetes risk calculator |  |  |
| 99822 | 38DK.00 | Finnish diabetes risk score |  |  |
| 107554 | 38Gv.00 | Diabetes UK diabetes risk score |  |  |
| NHS Health Check |  |  |  |  |
| 106237 | 9mC3.00 | NHS Health Check invitation third letter |  |  |
| 100682 | 9NiS.00 | Did not attend NHS Health Check |  |  |
| 105569 | 8BAg000 | NHS Health Check completed by third party |  |  |
| 106223 | 9mC4.00 | NHS Health Check verbal invitation |  |  |
| 106361 | 8IEd.00 | NHS Health Check annual review declined |  |  |
| 107476 | 9NSH.00 | NHS Health Check not appropriate |  |  |
| 107003 | 9RL0.00 | NHS continuing healthcare checklist completed |  |  |
| 110462 | 9mC6.00 | NHS Health Check invitation SMS text message |  |  |
| 106222 | 9mC0.00 | NHS Health Check telephone invitation |  |  |
| 102360 | 6AH..00 | NHS Health Check annual review |  |  |
| 106215 | 9mC..00 | NHS Health Check invitation |  |  |
| 100460 | 9Nj5.00 | Failed to respond to NHS Health Check invitation |  |  |
| 99856 | 8BAg.00 | NHS Health Check completed |  |  |
| 102511 | 8IAx.00 | NHS Health Check declined |  |  |
| 106961 | 9Nie.00 | Did not attend NHS Health Check annual review |  |  |
| 106217 | 9mC1.00 | NHS Health Check invitation first letter |  |  |
| 100000 | 8BR2.00 | NHS Health Check indicated |  |  |
| 106221 | 9mC2.00 | NHS Health Check invitation second letter |  |  |
| 100142 | 6B5..00 | NHS Health Check programme |  |  |
| 108311 | 8HBR.00 | NHS Health Check follow up |  |  |
| 110509 | 9mC5.00 | NHS Health Check invitation email |  |  |
| Bacterial infections (UTI, LRTI, cellulitis) |  |  |  |  |
| 150 | K190z00 | urinary tract infection, site not specified nos |  |  |
| 389 | K15..00 | cystitis |  |  |
| 1289 | K190.00 | urinary tract infection, site not specified |  |  |
| 7579 | 1J4..00 | suspected uti |  |  |
| 10857 | K15y.00 | other specified cystitis |  |  |
| 12484 | K15z.00 | cystitis nos |  |  |
| 15074 | K150.00 | acute cystitis |  |  |
| 34630 | K15yz00 | other cystitis nos |  |  |
| 70189 | Kyu5100 | [x]other cystitis |  |  |
| 97002 | K190500 | urinary tract infection |  |  |
| 104141 | K190600 | urosepsis |  |  |
| medcode | readcode | readterm |  |  |
| 68 | H06z011 | chest infection |  |  |
| 312 | H060.00 | acute bronchitis |  |  |
| 572 | H26..00 | pneumonia due to unspecified organism |  |  |
| 886 | H25..00 | bronchopneumonia due to unspecified organism |  |  |
| 1382 | H060w00 | acute viral bronchitis unspecified |  |  |
| 1934 | H301.00 | laryngotracheobronchitis |  |  |
| 2581 | H06z000 | chest infection nos |  |  |
| 3163 | H300.00 | tracheobronchitis nos |  |  |
| 3358 | H06z100 | lower resp tract infection |  |  |
| 3480 | H30z.00 | bronchitis nos |  |  |
| 3683 | H261.00 | basal pneumonia due to unspecified organism |  |  |
| 5978 | H060.11 | acute wheezy bronchitis |  |  |
| 6094 | H2z..00 | pneumonia or influenza nos |  |  |
| 6124 | H062.00 | acute lower respiratory tract infection |  |  |
| 9389 | H20..11 | chest infection - viral pneumonia |  |  |
| 9639 | H260.00 | lobar pneumonia due to unspecified organism |  |  |
| 11072 | H060300 | acute purulent bronchitis |  |  |
| 11101 | H060500 | acute tracheobronchitis |  |  |
| 14976 | H20z.00 | viral pneumonia nos |  |  |
| 16287 | H25..11 | chest infection - unspecified bronchopneumonia |  |  |
| 17359 | H30..11 | chest infection - unspecified bronchitis |  |  |
| 19400 | H26..11 | chest infection - pnemonia due to unspecified organism |  |  |
| 20198 | H060z00 | acute bronchitis nos |  |  |
| 21061 | H3y0.00 | chronic obstruct pulmonary dis with acute lower resp infectn |  |  |
| 22795 | H22..11 | chest infection - other bacterial pneumonia |  |  |
| 23095 | H22z.00 | bacterial pneumonia nos |  |  |
| 23333 | H540000 | hypostatic pneumonia |  |  |
| 24356 | H540100 | hypostatic bronchopneumonia |  |  |
| 24800 | H060x00 | acute bacterial bronchitis unspecified |  |  |
| 28634 | H22..00 | other bacterial pneumonia |  |  |
| 30653 | H23..11 | chest infection - pneumonia organism os |  |  |
| 33478 | H20y.00 | viral pneumonia nec |  |  |
| 37447 | H06z112 | acute lower respiratory tract infection |  |  |
| 40498 | H24..00 | pneumonia with infectious diseases ec |  |  |
| 43884 | H22yz00 | pneumonia due to bacteria nos |  |  |
| 50867 | H22y.00 | pneumonia due to other specified bacteria |  |  |
| 52520 | Hyu0800 | [x]other viral pneumonia |  |  |
| 53753 | Hyu0H00 | [x]other pneumonia, organism unspecified |  |  |
| 63763 | Hyu0A00 | [x]other bacterial pneumonia |  |  |
| 66362 | H24z.00 | pneumonia with infectious diseases ec nos |  |  |
| 66397 | Hyu1.00 | [x]other acute lower respiratory infections |  |  |
| 69782 | H24y.00 | pneumonia with other infectious diseases ec |  |  |
| 70559 | H24yz00 | pneumonia with other infectious diseases ec nos |  |  |
| 98381 | Hyu0B00 | [x]pneumonia due to other specified infectious organisms |  |  |
| 104121 | H2B..00 | community acquired pneumonia |  |  |
| 4126 | A98yy14 | Gonococcal cellulitis |  |  |
| 4328 | F4G0100 | Orbital cellulitis |  |  |
| 8852 | F501112 | Cellulitis, external ear |  |  |
| 25156 | H1y2100 | Pharynx or nasopharynx cellulitis |  |  |
| 64484 | H1y5100 | Cellulitis of vocal cords |  |  |
| 61518 | H1y7100 | Cellulitis of larynx |  |  |
| 4748 | J083.00 | Oral cellulitis and abscess |  |  |
| 5228 | J083000 | Cellulitis of floor of mouth |  |  |
| 15336 | J083100 | Oral soft tissue cellulitis unspecified |  |  |
| 19944 | J083z00 | Oral cellulitis and abscess NOS |  |  |
| 17562 | J085100 | Cellulitis of lip |  |  |
| 27933 | J54..11 | Cellulitis - anus or rectum |  |  |
| 37424 | J540.11 | Perianal cellulitis |  |  |
| 26239 | K170300 | Periurethral cellulitis |  |  |
| 16304 | K272300 | Cellulitis of penis |  |  |
| 4456 | K284300 | Cellulitis of scrotum |  |  |
| 70783 | K403.00 | Acute parametritis and pelvic cellulitis |  |  |
| 33659 | K403100 | Acute pelvic cellulitis |  |  |
| 48663 | K404.00 | Chronic parametritis and pelvic cellulitis |  |  |
| 24294 | K404000 | Chronic female pelvic cellulitis |  |  |
| 15687 | K405.00 | Parametritis and pelvic cellulitis unspecified |  |  |
| 30982 | K405100 | Pelvic cellulitis unspecified |  |  |
| 5697 | M02..00 | Cellulitis and abscess of finger and toe |  |  |
| 4779 | M020.00 | Cellulitis and abscess of finger |  |  |
| 3527 | M020000 | Cellulitis and abscess of finger unspecified |  |  |
| 26071 | M020z00 | Cellulitis and abscess of finger NOS |  |  |
| 3960 | M021.00 | Cellulitis and abscess of toe |  |  |
| 3363 | M021000 | Cellulitis and abscess of toe unspecified |  |  |
| 20384 | M021z00 | Cellulitis and abscess of toe NOS |  |  |
| 25081 | M02z.00 | Cellulitis and abscess of digit NOS |  |  |
| 16536 | M03..00 | Other cellulitis and abscess |  |  |
| 16606 | M03..13 | Cellulitis of skin area excluding digits of hand or foot |  |  |
| 3998 | M030.00 | Cellulitis and abscess of face |  |  |
| 24401 | M030000 | Cellulitis and abscess of cheek (external) |  |  |
| 2658 | M030011 | Cellulitis and abscess of cheek |  |  |
| 21580 | M030100 | Cellulitis and abscess of nose (external) |  |  |
| 10485 | M030111 | Cellulitis and abscess of nose |  |  |
| 15549 | M030200 | Cellulitis and abscess of chin |  |  |
| 15327 | M030300 | Cellulitis and abscess of submandibular region |  |  |
| 15475 | M030400 | Cellulitis and abscess of forehead |  |  |
| 16032 | M030500 | Cellulitis and abscess of temple region |  |  |
| 27681 | M030600 | Cellulitis of face |  |  |
| 20389 | M030z00 | Cellulitis and abscess of face NOS |  |  |
| 2711 | M031.00 | Cellulitis and abscess of neck |  |  |
| 27717 | M032.00 | Cellulitis and abscess of trunk |  |  |
| 4394 | M032000 | Cellulitis and abscess of chest wall |  |  |
| 16176 | M032100 | Cellulitis and abscess of breast |  |  |
| 1874 | M032200 | Cellulitis and abscess of back |  |  |
| 4973 | M032300 | Cellulitis and abscess of abdominal wall |  |  |
| 14937 | M032400 | Cellulitis and abscess of umbilicus |  |  |
| 23585 | M032500 | Cellulitis and abscess of flank |  |  |
| 1923 | M032600 | Cellulitis and abscess of groin |  |  |
| 4400 | M032700 | Cellulitis and abscess of perineum |  |  |
| 52366 | M032800 | Cellulitis of trunk |  |  |
| 29345 | M084.00 | [X]Cellulitis of breast |  |  |
| 6368 | M085.00 | Cellulitis of leg |  |  |
| 31534 | M086.00 | Cellulitis of ankle |  |  |
| 9648 | M088.00 | Cellulitis of arm |  |  |
| 28181 | M089.00 | Cellulitis of neck |  |  |
| 17226 | M08A.00 | Cellulitis of axilla |  |  |
| 7684 | M08B.00 | Cellulitis of foot |  |  |
| 94868 | M08C.00 | Cellulitis of toe |  |  |
| 30260 | M08y.00 | [X]Cellulitis of other sites |  |  |
| Structured diabetes programme |  |  |  |  |
| 93854 | 9OLM.00 | Diabetes structured education programme declined |  |  |
| 26605 | 9OLB.00 | Attended diabetes structured education programme |  |  |
| 47011 | 8Hj0.00 | Referral to diabetes structured education programme |  |  |
| 107414 | 8I94.00 | Diabetes structured education programme not available |  |  |
| 95093 | 8I83.00 | Did not complete DESMOND diabetes structured educat program |  |  |
| 94956 | 8I84.00 | Did not complete XPERT diabetes structured education program |  |  |
| 97809 | 8I82.00 | Did not complete DAFNE diabetes structured education program |  |  |
| 95641 | 8Hj1.00 | Family/carer referral to diabetes structured education prog |  |  |
| 94955 | 9NiE.00 | Did not attend XPERT diabetes structured education programme |  |  |
| 93631 | 9OLL.00 | XPERT diabetes structured education programme completed |  |  |
| 110511 | 67W1.00 | Recommendation self-refer for diabetes structured education |  |  |
| 106953 | 8IEa.00 | Referral to DAFNE diabetes structured educn prog declined |  |  |
| 95094 | 8I81.00 | Did not complete diabetes structured education programme |  |  |
| 93491 | 9OLJ.00 | DAFNE diabetes structured education programme completed |  |  |
| 95159 | 9NiD.00 | Did not attend DESMOND diabetes structured education program |  |  |
| 93870 | 8Hj5.00 | Referral to XPERT diabetes structured education programme |  |  |
| 94011 | 9OLG.00 | Attended XPERT diabetes structured education programme |  |  |
| 99277 | 9NiC.00 | Did not attend DAFNE diabetes structured education programme |  |  |
| 93657 | 8Hj4.00 | Referral to DESMOND diabetes structured education programme |  |  |
| 12682 | 679R.00 | Patient offered diabetes structured education programme |  |  |
| 51066 | 9OLC.00 | Family/carer attended diabetes structured education prog |  |  |
| 93390 | 9OLH.00 | Attended DAFNE diabetes structured education programme |  |  |
| 94186 | 9OLF.00 | Diabetes structured education programme completed |  |  |
| 93704 | 8Hj3.00 | Referral to DAFNE diabetes structured education programme |  |  |
| 95553 | 9NiA.00 | Did not attend diabetes structured education programme |  |  |
| 93529 | 9OLK.00 | DESMOND diabetes structured education programme completed |  |  |
| Diabetes review |  |  |  |  |
| 6125 | 66AS.00 | Diabetic annual review |  |  |
| 11471 | 8B3l.00 | Diabetes medication review |  |  |
| 28873 | 66Ai.00 | Diabetic 6 month review |  |  |
| 32619 | 66Af.00 | Patient diabetes education review |  |  |
| 83532 | 66Ao.00 | Diabetes type 2 review |  |  |
| 101177 | 66At.00 | Diabetic dietary review |  |  |
| 101801 | 66At100 | Type II diabetic dietary review |  |  |
| 102434 | 66Au.00 | Diabetic erectile dysfunction review |  |  |
| 102611 | 66At111 | Type 2 diabetic dietary review |  |  |
| 107423 | 661N400 | Diabetes self-management plan review |  |  |
| 107464 | 66AS000 | Diabetes Year of Care annual review |  |  |
| Retinopathy screening/review |  |  |  |  |
| 18662 | 8HBH.00 | Diabetic retinopathy 6 month review |  |  |
| 18311 | 68A7.00 | Diabetic retinopathy screening |  |  |
| 11891 | 68A8.00 | Digital retinal screening |  |  |
| 9974 | 9N1v.00 | Seen in diabetic eye clinic |  |  |
| 12636 | 9N2f.00 | Seen by retinal screener |  |  |
| 18747 | 8I6F.00 | Diabetic retinopathy screening not indicated |  |  |
| 12262 | 8I3X.00 | Diabetic retinopathy screening refused |  |  |
| Diabetic foot screening |  |  |  |  |
| 22823 | 66Ab.00 | Diabetic foot examination |  |  |
| 95994 | 66Aq.00 | Diabetic foot screen |  |  |
| 50175 | 66AW.00 | Diabetic foot risk assessment |  |  |
| 108890 | 679L300 | Diabetic foot care education |  |  |
| 18824 | 8I3W.00 | Diabetic foot examination declined |  |  |
| 12247 | 8I6G.00 | Diabetic foot examination not indicated |  |  |
| 10824 | 9N1i.00 | Seen in diabetic foot clinic |  |  |

Table S5. Code lists
